# Multi-Gene Phylogeny and Taxonomy of the Wood-Rotting Fungal Genus *Phlebia sensu lato* (Polyporales, Basidiomycota)

**DOI:** 10.3390/jof9030320

**Published:** 2023-03-05

**Authors:** Changlin Zhao, Menghan Qu, Ruoxia Huang, Samantha C. Karunarathna

**Affiliations:** 1 Yunnan Key Laboratory of Plateau Wetland Conservation, Restoration and Ecological Services, Southwest Forestry University, Kunming 650224, China; 2 Yunnan Key Laboratory for Fungal Diversity and Green Development, Kunming Institute of Botany, Chinese Academy of Sciences, Kunming 650201, China; 3 College of Biodiversity Conservation, Southwest Forestry University, Kunming 650224, China; 4 College of Forestry, Southwest Forestry University, Kunming 650224, China; 5 Center for Yunnan Plateau Biological Resources Protection and Utilization, College of Biological Resource and Food Engineering, Qujing Normal University, Qujing 655011, China

**Keywords:** biodiversity, degradation, Meruliaceae, molecular systematics, wood-inhabiting fungi

## Abstract

*Phlebia* s.l. (Polyporales, Basidiomycota) accommodates numerous species of wood-inhabiting fungi within the phylum Basidiomycota. The present study employs the morphological and phylogenetic approaches to revise the generic and species classification of *Phlebia* s.l. and surveys the species diversity. The phylogenetic analyses were performed using multiple gene regions viz. the internal transcribed spacer (ITS), the large subunit nuclear ribosomal RNA gene (nLSU), the translation elongation factor 1-α (tef1), the small subunit of mitochondrial rRNA gene (mtSSU), the glyceraldehyde 3-phosphate dehydrogenase (GAPDH), RNA polymerase II largest subunit (rpb1), and RNA polymerase II second largest subunit (rpb2). We overall recognize twenty genera of *Phlebia* s.l., including three new genera viz. *Ceriporiopsoides*, *Phlebicolorata*, and *Pseudophlebia*, seven new species viz. *Crustodontia rhododendri*, *Hydnophlebia fissurata*, *Luteoporia straminea*, *Merulius sinensis*, *Mycoaciella brunneospina*, *Phlebia niveomarginata*, and *P. poroides* and seventeen new combinations viz. *Ceriporiopsoides guidella*, *C. lagerheimii*, *Hydnophlebia acanthocystis*, *H. capsica*, *H. fimbriata*, *Merulius fuscotuberculatus*, *M. nantahaliensis*, *M. tomentopileatus*, *Mycoacia tuberculata*, *Mycoaciella uda*, *Phlebicolorata alboaurantia*, *Ph. brevispora*, *Ph. pseudoplacenta*, *Ph. rosea*, *Pseudophlebia lindtneri*, *Ps. semisupina*, and *Ps. setulosa*. Descriptions, illustrations, phylogenetic trees to show the placements, and notes of new taxa are provided.

## 1. Introduction

Taxonomy plays a significant role in revealing the diversity and classification of life and the discovery of specimens and observations into systems of names, in which it captures the relationships among taxa [1]. Fungi play a diverse and ecologically important role in the tree of life, in which the organisms exist in ecosystems mainly on wood, soil, leaves, rocks, and pelagic zones of the ocean [2]. Wood-rotting fungi are a cosmopolitan fungal group with a rich diversity in boreal, temperate, subtropical, and tropical vegetations, in which they degrade hard-to-digest substrates, such as lignin, cellulose, and pollen to push the sustainable ecosystem cycle [3,4]. The fungal order Polyporales Gäum. is a core group of the wood-rotting fungi located in the class Agaricomycetes Doweld (Basidiomycota R.T. Moore), which includes about 2500 species [5]. The family Meruliaceae Rea includes 21 genera viz. *Aurantiopileus* Ginns, D. L. Lindner & T.J. Baroni, *Aurantiporus* Murrill, *Ceriporiopsis* Domański, *Climacodon* P. Karst., *Crustodontia* Hjortstam & Ryvarden, *Geesterania* Westphalen, Tomšovský & Rajchenb., *Hermanssonia* Zmitr., *Hydnophanerochaete* Sheng H. Wu & C.C. Chen, *Hydnophlebia* Parmasto, *Lilaceophlebia* (Parmasto) Spirin & Zmitr., *Luteoporia* F. Wu, Jia J. Chen & S.H. He, *Merulius* Fr., *Mycoacia*, *Mycoaciella*, *Odoria* V. Papp & Dima, *Pappia* Zmitr., *Phlebia* Fr., *Phlebiporia* Jia J. Chen, B.K. Cui & Y.C. Dai, *Sarcodontia* Schulzer, *Scopuloides* (Massee) Höhn. & Litsch., and *Stereophlebia* Zmitr. [6], in which the genus *Phlebia* is closely related to the type genus *Merulius* (Meruliaceae) and acts as a core group in this family [7,8,9,10,11,12,13,14,15,16,17,18,19,20,21].

The genus *Phlebia* Fr. (Meruliaceae, Polyporales), erected by Fries [8] with *P. radiata* Fr. as the type species, is a large, cosmopolitan genus characterized by the effused or partly pileate basidiomata with a subceraceous to subgelatinous texture when fresh, membranaceous to coriaceous when dry, hymenophore smooth, tuberculate, phlebioid, odontioid, merulioid or poroid, a monomitic (rarely dimitic) hyphal structure generally with clamped hyphae, the embedded generative hyphae very difficult to observe, narrowly clavate basidia, and colorless, thin-walled, smooth, allantoid to ellipsoid basidiospores, which are acyanophilous, in-amyloid and non-dextrinoid [7,22,23,24,25,26,27,28,29,30,31,32,33]. Currently, about 100 species have been accepted in the genus worldwide [34,35,36].

Recently, mycologists employed molecular data on the genus *Phlebia sensu lato* to establish a phylogenetic frame for the classification of this genus, which indicates that *Phlebia* s.l. is polyphyletic [37,38]. Earlier, molecular systematics placed *Phlebia* in the polyporoid clade [39], and later the phylogenetic research among corticioid homobasidiomycetes suggested that the genus *Phlebia* should be located in the phlebioid clade with related genera *Ceriporia* Donk and *Gloeoporus* Mont. Additionally, the phlebioid clade was divided into three subclades that were interpretable also in terms of morphology, which indicated that *Phlebia* s. str., *Mycoacia*, and *Mycoaciella* were mainly referred to *Phlebia* s.l. [40]. Larsson [37] studied the classification of corticioid fungi, which revealed that *Phlebia* clusters into the family *Meruliaceae* within the order Polyporales. Mycologists focused on the phylogenetic study of European *Ceriporiopsis* Domański taxa, which revealed that *Phlebia radiata* and *C. gilvescens* (Bres.) Domański grouped together at the base of the combined data of the large subunit nuclear ribosomal RNA gene (nLSU) sequences and mitochondrial small subunit rRNA (mtSSU) gene sequences, but the taxa between *Phlebia* and *Ceriporiopsis* were left to be resolved in the future [41]. Justo et al. revised the family-level classification of the order Polyporales by using a multigene dataset, which showed that *Phlebia radiata* belonged to the family *Meruliaceae* and grouped with related genera *Aurantiporus* Murrill and *C. gilvescens*, in which the species of *Phlebia* s.l. were found in three different families: Phanerochaetaceae, Irpicaceae, and Meruliaceae; therefore, it was suggested that extensive molecular sampling was essential to establish sound generic concepts in *Phlebia* s.l., based on a combination of morphological features and molecular evidence [38]. Huang et al. have run a phylogenetic analysis, which showed that *Phlebia* species clustered into phlebioid clade with three new *Phlebia* species viz. *P. fuscotuberculata*, *P. tomentopileata,* and *P. tongxiniana* from southern China [32].

Recently, *Phlebia* s.l. has been intensively studied based on phylogenetic analyses using the multi-gene regions [34,41,42]. Species delimitation within the genus *Phlebia* s.l. is still not settled, and some members of the genus are scattered in different lineages [27,33,34,38], which are not fully consistent with the morphological features, such as *Ceriporiopsis gilvescens* (Bres.) Domański [43], *C. guidella* Bernicchia & Ryvarden and *C. lagerheimii* Læssøe & Ryvarden [44], and *P. setulosa* (Berk. & M.A. Curtis) Nakasone [34]. Furthermore, there are still abundant new species required to be found and reported, while numerous molecular sequences are lacking for many known species. Thus, it is essential to provide a comprehensive investigation of *Phlebia* s.l. based on multi-gene phylogenetic analyses.

This study aims to establish a phylogenetic overview of the genus *Phlebia* s.l., according to the morphological and multi-gene phylogenetic analyses of abundant known species and numerous novel taxa. Three new genera, seven new species, and 17 new combinations are proposed in this study.

## 2. Materials and Methods

### 2.1. Sample Collection and Herbarium Specimen Preparation

Fresh fruiting bodies of the fungi growing on the angiosperm stump, on the stump of angiosperm were collected from the Honghe of Yunnan Province, China. The samples were photographed in situ, and fresh macroscopic details were recorded [31]. Photographs were recorded by a Jianeng 80D camera. All of the photos were focus-stacked and merged using Helicon Focus software. Macroscopic details were recorded and transported to a field station where the fruit body was dried on an electronic food dryer at 45 °C. Once dried, the specimens were sealed in an envelope and zip lock plastic bags and labeled [44]. The dried specimens were deposited in the herbarium of the Southwest Forestry University (SWFC), Kunming, Yunnan Province, China.

### 2.2. Morphology

Macromorphological descriptions are based on field notes and photos captured in the field and lab. Micromorphological data were obtained from the dried specimens following observation under a light microscope [31]. The following abbreviations were used: KOH = 5% potassium hydroxide water solution, CB = cotton clue, CB– = acyanophilous, IKI = Melzer’s reagent, IKI– = both inamyloid and indextrinoid, L = means spore length (arithmetic average for all spores), W = means spore width (arithmetic average for all spores), Q = variation in the L/W ratios between the specimens studied, and n = a/b (number of spores (a) measured from a given number (b) of specimens).

### 2.3. DNA Extraction and Sequencing

The EZNA HP Fungal DNA Kit (Omega Biotechnologies Co., Ltd., Kunming, China) was used to extract DNA from the dried specimens, according to the manufacturer’s instructions, with some modifications. The ITS region was amplified with the primer pair ITS5/ITS4 [45], the nLSU region with the primer pair LR0R/LR7 [46], the TEF1 region with the primer pair EF1-983F/EF1-2218R [47], the mt-SSU region with the primer pair MS1/MS2 [45], the GAPDH region with the primer pair GAPDH-F/GAPDH-R [48], the RPB1 region with the primer pair RPB1-Af/RPB1-Cf [49], and the RPB2 region with the primer pair bRPB2-6F/bRPB2-7.1R [50]. The primer sets used for nucleotide PCR amplification, and the sequences are listed in Table 1. The PCR procedure for ITS was as follows: initial denaturation at 95 °C for 3 min, followed by 35 cycles at 94 °C for 40 s, 58 °C for 45 s and 72 °C for 1 min, and a final extension of 72 °C for 10 min. The PCR procedure for nLSU was as follows: initial denaturation at 94 °C for 1 min, followed by 35 cycles at 94 °C for 30 s, 48 °C for 1 min and 72 °C for 1.5 min, and a final extension of 72 °C for 10 min. The PCR procedure for TEF1 was as follows: (1) initial denaturation at 94 °C for 2.5 min, (2) denaturation at 94 °C for 45 s, (3) annealing at 60 °C for 50 s (minus 1 C per cycle), (4) extension at 72 °C for 2 min, (5) repeat for 6 cycles starting at step 2, (6) denaturation at 94 °C for 30 s, (7) annealing at 55 °C for 50 s, (8) extension at 72 °C for 1.5 min, (9) repeat for 34 cycles starting at step 6, (10) leave at 72 °C for 5 min. The PCR procedure for mt-SSU was as follows: initial denaturation at 94 °C for 2 min, followed by 36 cycles at 94 °C for 45 s, 52 °C for 45 s and 72 °C for 1 min, and a final extension of 72 °C for 10 min. The PCR procedure for GAPDH was as follows: initial denaturation at 95 °C for 3 min, followed by 35 cycles at 94 °C for 40 s, 50 °C for 45 s and 72 °C for 1 min, and a final extension of 72 °C for 10 min. The PCR procedure for RPB1 was as follows: (1) initial denaturation at 94 °C for 2 min, (2) denaturation at 94 °C for 40 s, (3) annealing at 60 °C for 40 s, (4) extension at 72 °C for 2 min, (5) repeat for 10 cycles starting at step 2, (6) denaturation at 94 °C for 45 s, (7) annealing at 55 °C for 1.5 min, (8) extension at 72 °C for 2 min, (9) repeat for 37 cycles starting at step 6, (10) leave at 72 °C for 10 min. The PCR procedure for RPB2 was as follows: (1) initial denaturation at 95 °C for 2.5 min, (2) denaturation at 95 °C for 30 s, (3) annealing at 52 °C for 1 min, (4) extension at 72 °C for 1 min (add 1 C per cycle), (5) repeat for 40 cycles starting at step 2, (6) extension at 72 °C for 1.5 min, (7) repeat for 40 cycles starting at step 6, (8) leave at 72 °C for 5 min. The PCR products were purified and directly sequenced at Kunming Tsingke Biological Technology Limited Company, Yunnan Province, China. All of the newly generated sequences were deposited in GenBank (Table 2).

### 2.4. Phylogenetic Analyses

**Figure 1:**
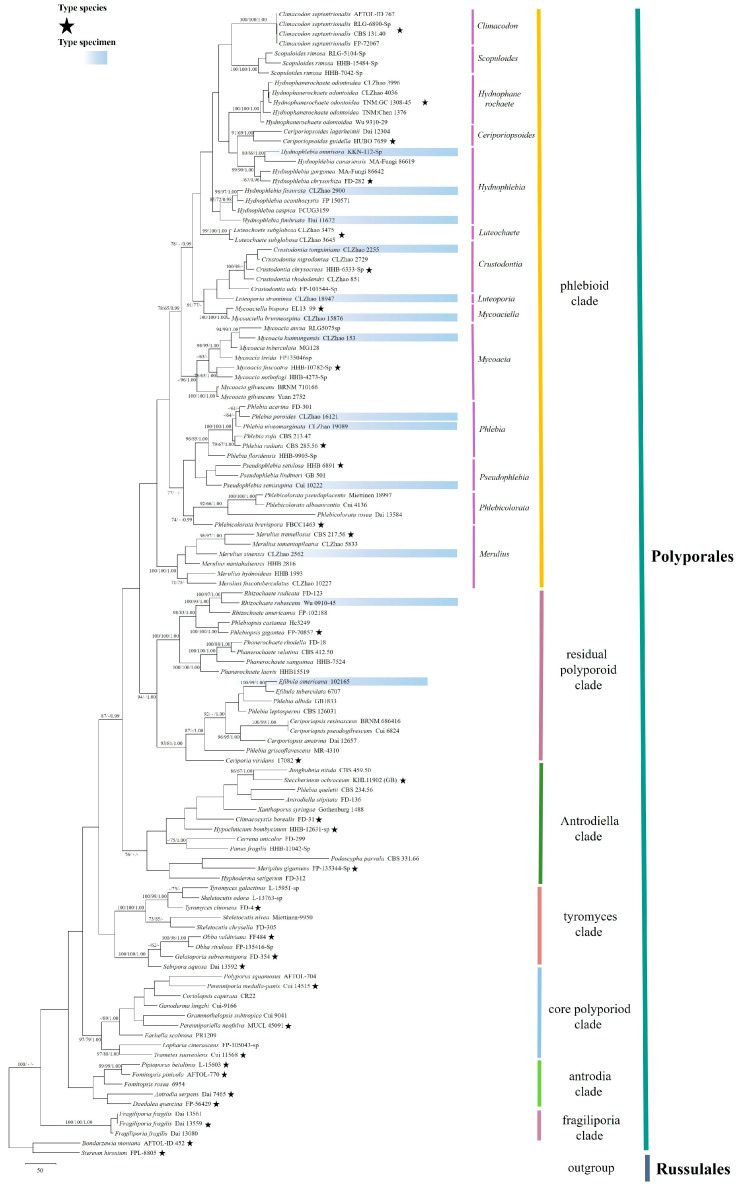
Maximum Parsimony strict consensus tree illustrating the phylogeny of *Phlebia* species and related genera in the order Polyporales based on ITS + nLSU sequences. Branches are labeled with BS > 70%, BT > 50% and BPP > 0.95, respectively. Clade names follow the previous study by Justo et al. [38]. The asterisks represent the type species.

**Figure 2:**
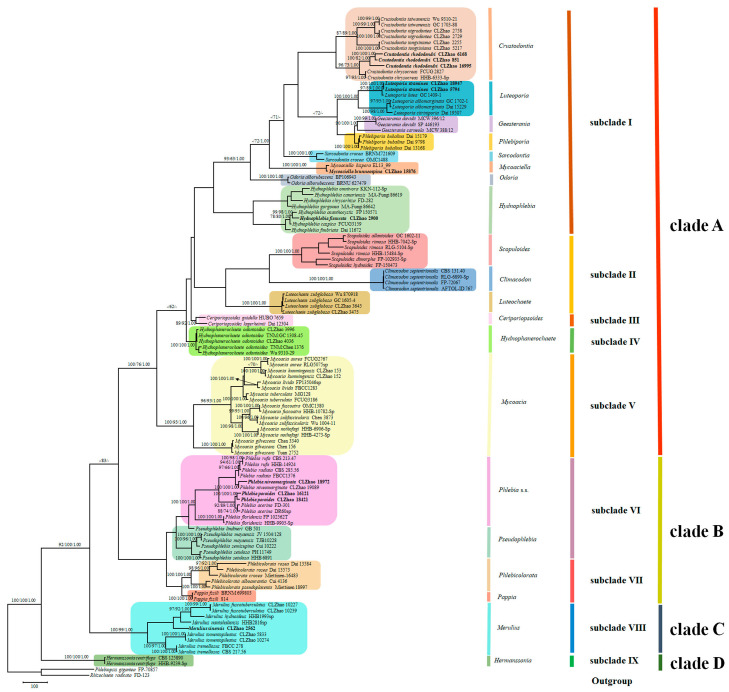
Maximum Parsimony strict consensus tree illustrating the phylogeny of *Phlebia* in the family Meruliaceae based on ITS + nLSU + TEF1 + mt-SSU + GAPDH + RPB1 + RPB2 sequences. Branches are labeled with (BS) > 70%, (BT) > 50% and (BPP) > 0.95, respectively. The new species are in bold.

Sequencher 4.6 (GeneCodes, Ann Arbor, MI, USA) was used to edit the DNA sequence chromatograms. The sequences were aligned in MAFFT 7 (https://mafft.cbrc.jp/alignment/server/ (accessed on 7 August 2022)) using the “G-INS-i” strategy and adjusted manually in BioEdit [80]. The sequence alignments were deposited in TreeBase (ID 28428; http://purl.org/phylo/treebase/phylows/study/TB2:S28428?x-access-code=c213567340d8eaabcc76d6421c07840d&format=html (accessed on 15 August 2022)). (1) *Bondarzewia montana* (Quél.) Singer and *Stereum hirsutum* (Willd.) Pers. were assigned as an outgroup to root trees following Floudas & Hibbett [28] in the ITS + nLSU analysis (Figure 1); (2) *Phlebiopsis gigantea* (Fr.) Jülich and *Rhizochaete radicata* (Henn.) Gresl., Nakasone & Rajchenb were used as an outgroup to root trees following Justo et al. [38] in the ITS + nLSU + TEF1 + mt-SSU + GAPDH + RPB1 + RPB2 analyses (Figure 2).

Maximum parsimony analysis was applied to the combined dataset and followed Zhao and Wu [64]; the tree construction procedure was performed in PAUP * version 4.0b10 [81]. All of the characters were equally weighted, and the gaps were treated as missing data. Trees were inferred using the heuristic search option with TBR branch swapping and 1000 random sequence additions. Max-trees were set to 5000, branches of zero length were collapsed, and all parsimonious trees were saved. Clade robustness was assessed using a bootstrap (BT) analysis with 1000 replicates [82]. Descriptive tree statistics tree length (TL), consistency index (CI), homoplasy index (HI), retention index (RI), and rescaled consistency index (RC) were calculated for each Maximum Parsimonious Tree (MPT) generated. Ready datasets were also analyzed using Maximum Likelihood (ML) with RAxML-HPC2 software through the Cipres Science Gateway (www.phylo.org (accessed on 10 August 2022)) [83]. Branch support (BS) for ML analysis was determined by 1000 bootstrap replicates.

MrModeltest 2.3 [84] was used to determine the best-fit evolution model for each data set for Bayesian inference (BI) of the phylogeny. Bayesian inference was calculated with MrBayes 3.1.2 [85]. Four Markov chains were run for 2 runs from random starting trees for 5 million generations for the first dataset (Figure 1), for 2 million generations for the second dataset (Figure 2), and the trees were sampled every 100 generations; the first one-fourth of generations were discarded as burn-in. A majority-rule consensus tree of all remaining trees was calculated. Branches were considered as significantly supported if they received maximum likelihood bootstrap (BS) >70%, maximum parsimony bootstrap (BT) >50%, or Bayesian posterior probabilities (BPP) >0.95.

## 3. Results

### 3.1. Phylogenetic Analyses

In this study, 16 specimens belonging to *Phlebia* s.l. were newly examined and sequenced. From these 16 specimens, we generated one ITS, one nLSU, nine TEF1α, eleven RPB1, eight RPB2 and nine GAPDH sequences (Table 2). 

The first combined ITS + nLSU dataset (Figure 1) comprises sequences from 117 specimens and 105 species in Polyporales. The dataset had an aligned length of 1830 characters, of which 1019 characters were constant, 184 were variable and parsimony-uninformative, and 627 were parsimony-informative. Maximum parsimony analysis yielded 5000 equally parsimonious trees (TL = 7191, CI = 0.1972, HI = 0.8028, RI = 0.5284, RC = 0.1042). The best model suggested by MrModeltest and applied in BI was GTR + I + G. Bayesian analysis and ML analysis resulted in a similar topology as MP analysis; BI had the average standard deviation of split frequencies = 0.008528 (BI), and the effective sample size (ESS) across the two runs is double that of the average ESS (avg ESS) = 479. The phylogenetic tree inferred from ITS + nLSU sequences (Figure 1) demonstrated seven major clades, the antrodia clade, core polyporoid clade, fragiliporia clade, gelatoporia clade, phlebioid clade, residual polyporoid clade, and the tyromyces clade, for 106 sampled species in Polyporales. The *Phlebia* s.l. species belonged to the phlebioid clade, in which *Phlebia* s.s. grouped with *Merulius*, *Phlebicolorata*, and *Pseudophlebia* with lower supports.

The second dataset based on ITS + nLSU + TEF1 + mt-SSU + GAPDH + RPB1 + RPB2 (Figure 2) comprises sequences from 113 specimens and 65 species within the family Meruliaceae. The dataset had an aligned length of 5683 characters, of which 3061 characters were constant, 714 were variable and parsimony-uninformative, and 1908 were parsimony-informative. Maximum parsimony analysis yielded 48 equally parsimonious trees (TL = 10,321, CI = 0.4137, HI = 0.5863, RI = 0.6182, RC = 0.2557). Bayesian analysis and ML analysis resulted in a similar topology as MP analysis; BI had the average standard deviation of split frequencies = 0.007583 (BI), and the effective sample size (ESS) across the two runs is double the average ESS (avg ESS) = 194. The phylogeny reconstruction (Figure 2) demonstrated that twenty genera, *Ceriporiopsoides*, *Climacodon*, *Crustodontia* Hjortstam & Ryvarden, *Geesterania* Westphalen, Tomšovský & Rajchenberg, *Hermanssonia* Zmitr., *Hydnophanerochaete* Sheng H. Wu & C.C. Chen, *Hydnophlebia*, *Luteochaete* C.C. Chen & Sheng H. Wu, *Luteoporia* F. Wu, Jia J. Chen & S.H. He, *Merulius*, *Mycoacia*, *Mycoaciella*, *Odoria* V. Papp & Dima, *Pappia* Zmitr., *Phlebia* s.s., *Phlebiporia* Jia J. Chen, B.K. Cui & Y.C. Dai, *Phlebicolorata*, *Pseudophlebia*, *Sarcodontia* Schulzer, and *Scopuloides* were included, in which ten clades were formed to cover taxa of *Phlebia* s.l. within the family Meruliaceae. Clade A includes *Ceriporiopsoides*, *Climacodon*, *Crustodontia*, *Geesterania*, *Hydnophlebia*, *Hydnophanerochaete*, *Luteochaete*, *Luteoporia*, *Mycoacia Mycoaciella*, *Odoria*, *Phlebiporia*, *Sarcodontia*, and *Scopuloides*, in which Subclade I comprises *Crustodontia*, *Geesterania*, *Hydnophlebia*, *Luteoporia*, *Mycoaciella*, *Odoria*, *Phlebiporia*, *Sarcodontia*; Subclade II comprises *Climacodon*, *Luteochaete* and *Scopuloides*; Subclade III comprises *Ceriporiopsoides*; Subclade IV comprises *Hydnophanerochaete*; Subclade V comprises *Mycoacia*. Clade B includes *Pappia*, *Phlebia* s.s., *Phlebicolorata,* and *Pseudophlebia*, in which Subclade VI comprises *Phlebia* s.s. and *Pseudophlebia*; Subclade VII comprises *Pappia* and *Phlebicolorata*. Clade C includes *Merulius*. Clade D includes *Hermanssonia*.

### 3.2. Taxonomy

***Ceriporiopsoides*** C.L. Zhao, gen. nov.

MycoBank: MB 843309.

Diagnosis: It is characterized by annual, resupinate, hard, brittle, cartilaginous basidiomata with a poroid hymenophore, a monomitic hyphal system with clamp connections and cylindrical, colorless, thin-walled, smooth basidiospores.

Index Fungorum number: IF843309; Facesoffungi number: FoF12678.

**Type species**—*Ceriporiopsoides guidella* (Bernicchia & Ryvarden) C.L. Zhao.

**Etymology**—Referring to the poroid hymenophore similar to *Ceriporiopsis*.

Basidiomata annual, resupinate, hard, brittle, cartilaginous. Hymenophore poroid, pore round to angular. Hyphal system monomitic; generative hyphae clamped, colorless, IKI–, CB–. Lack of cystidia and absence of crystals. Basidiospores are cylindrical, colorless, thin-walled, smooth, IKI–, CB–. White rot. It mainly differs from the other genus in terms of DNA sequences.

***Ceriporiopsoides guidella*** (Bernicchia & Ryvarden) C.L. Zhao, comb. nov.

MycoBank: MB 843310.

Index Fungorum number: IF843310; Facesoffungi number: FoF12676.

**Basionym**—*Ceriporiopsis guidella* Bernicchia & Ryvarden, Mycotaxon 88: 220. 2003.

**Notes**—Morphologically, it was originally described under the genus *Ceriporiopsis* based on the character of the hard, brittle basidiomata with the cracking pore surface, a monomitic hyphal system with the clamped generative hyphae, a lack of cystidia and the absence of crystals, and cylindrical, colorless, thin-walled, smooth basidiospores. However, it forms a monophyletic lineage based on the molecular evidence in the previous studies [31,34,44] as well as the present study (Figure 2), and we propose it as a generic species of the new genus *Ceriporiopsoides*.

***Ceriporiopsoides lagerheimii*** (Læssøe & Ryvarden) C.L. Zhao, comb. nov.

MycoBank: MB 843311.

Index Fungorum number: IF843311; Facesoffungi number: FoF12677.

**Basionym**—*Ceriporiopsis lagerheimii* Læssøe & Ryvarden, Syn. Fung. (Oslo) 27: 44. 2010.

**Notes**—This species was found in Napo Province of Ecuador on the underside of a trunk of Alnus, and it was settled into *Ceriporiopsis* based on a monomitic hyphal system with the clamped generative hyphae, lack of cystidia and absence of the crystals and colorless, thin-walled, smooth basidiospores. In the present study, it groups with *Ceriporiopsoides guidella*, belonging to the genus *Ceriporiopsoides* (Figure 2).

***Crustodontia*** Hjortstam & Ryvarden, Syn. Fung. (Oslo) 20: 36. 2005.

**Type species**—*Crustodontia chrysocreas* (Berk. & M.A. Curtis) Hjortstam & Ryvarden, Syn. Fung. (Oslo) 20: 36. 2005.

Basidiomata resupinate is ceraceous to subceraceous. Hymenophore is grandinoid to odontioid or tuberculata, yellowish, brownish, or black hymenial surface, turning to reddish or purplish with KOH. The hyphal system is monomitic, having generative hyphae with clamp connections. Cystidia is cylindrical to ventricose. Basidia clavate has four sterigmata. Basidiospores ellipsoid to broadly ellipsoid, thin-walled, smooth, IKI–, CB– [86].

**Key to species of *Crustodontia***

1. Hymenial surface cracking                       2

1. Hymenial surface not cracking                    3

2. Basidiospores >3 µm in wideth              *C. taiwanensis*

2. Basidiospores <3 µm in wideth              *C. rhododendri*

3. Hymenophore brown to black              *C. nigrodontea*

3. Hymenophore buff to ochraceous-buff to buckthorn brown       4

4. Basidiospores cylindrical, <2.5 µm in wideth        *C. chrysocreas*

4. Basidiospores ellipsoid, >2.5 µm in wideth         *C. tongxiniana*

**Figure 3:**
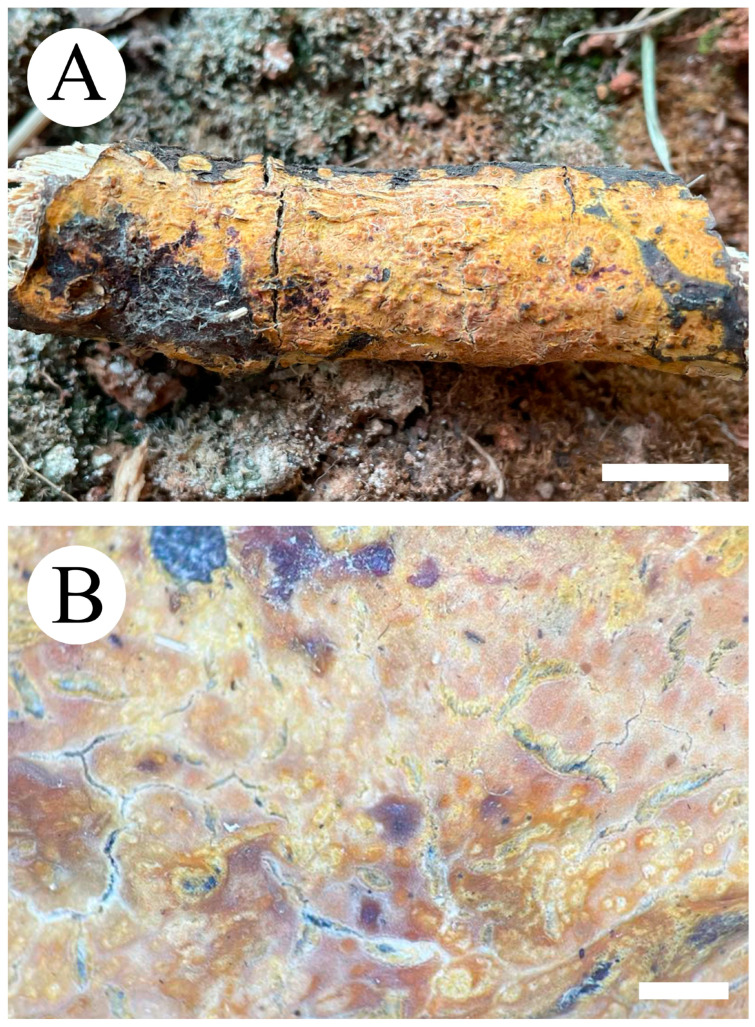
Basidioma of *Crustodontia rhododendri* (CLZhao 6168, holotype). Bars: (**A**) = 1 cm, (**B**) = 1 mm.

**Figure 4:**
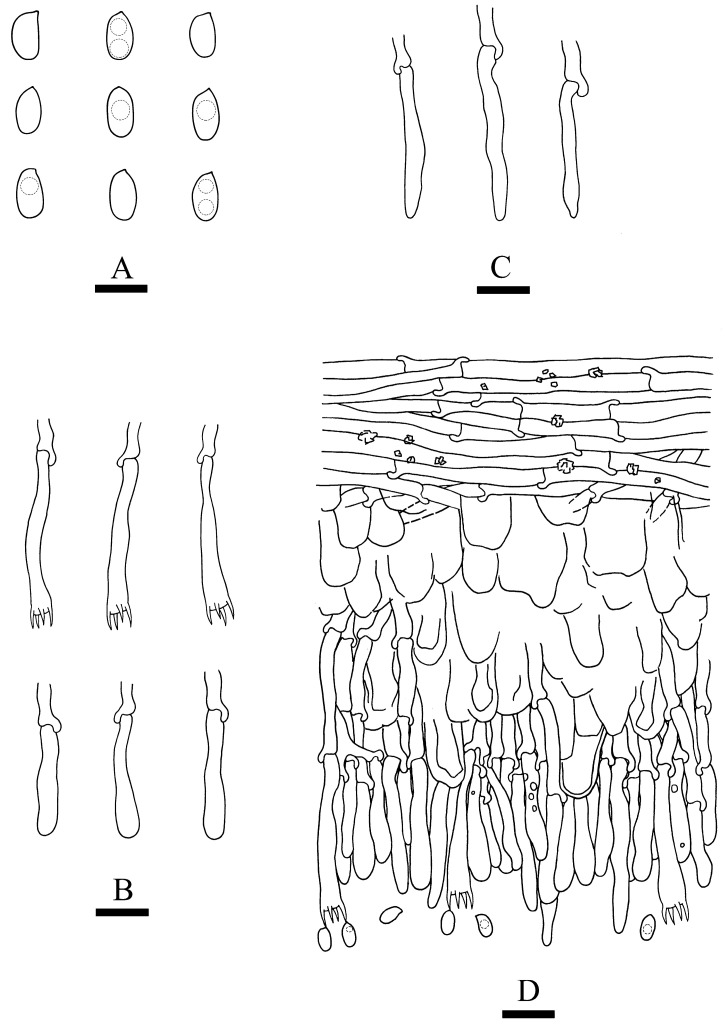
Microscopic structures of *Crustodontia rhododendri* (drawn from CLZhao 6168, holotype). (**A**) Basidiospores. (**B**) Basidia and basidioles. (**C**) Cystidioles. (**D**) A section of hymenium. Bars: (**A**) = 5 μm, (**B**–**D**) = 10 μm.

***Crustodontia rhododendri*** C.L. Zhao, sp. nov. (Figure 3 and Figure 4).

MycoBank: MB 843312.

Index Fungorum number: IF843312; Facesoffungi number: FoF12679.

Diagnosis: It differs from *C. taiwanensis* by tuberculate hymenophore with a straw to ochreous hymenial surface, slightly ochreous, fimbriate sterile margin, and narrower basidiospores measuring 3.7–5.2 × 1.9–2.9 µm.

**Holotype**—China, Yunnan Province, Puer, Zhenyuan County, Damoshan, E 101°37′, N 24°19′, alt. 1900 m, on fallen branch of *Rhododendron simii*, 15 January 2018, C.L. Zhao, CLZhao 6168 (SWFC).

**Etymology**—Referring to the host of *Rhododendron simii*.

Basidiomata annual, resupinate, ceraceous, without odor or taste when fresh, becoming coriaceous upon drying, up to 5.5 cm long, 2 cm wide, 100–300 µm thick. Hymenial surface tuberculate, buff when fresh, straw to ochreous upon drying, cracking, turning to reddish or purplish immediately with KOH. The sterile margin is narrow, 1 mm wide, slightly ochreous, fimbriate. Hyphal structure monomitic; generative hyphae clamped, colorless, thin-walled, unbranched, IKI–, CB–; tissues unchanged in KOH. Subicular hyphae subparallel, 3.5–5.5 µm in diameter; the subhymenial hyphae is unbranched, 2–5 µm in diameter; the presence of numerous yellow to yellowish brown gelatinous substances between subiculum and subhymenium. *Hymenium* cystidia cylindrical to ventricose, colorless, thin-walled, 17.5–40 × 2.7–5.4 µm; basidia cylindrical, with four sterigmata and a basal clamp connection, 10.3–33.4 × 3.3–5.6 µm. *Basidiospores* ellipsoid, colorless, thin-walled, smooth, often with 1–oil drops, IKI–, CB–, (35–)3.7–5.2(–5.5) × 1.9–2.9(–3.1) µm, L = 4.25 µm, W = 2.37 µm, Q = 1.68–1.87 (n = 360/12). 

**Distribution and ecology**—The species is known from southern China, growing in subtropical evergreen broad-leaved forests and has a white rot. 

**Specimens examined (paratypes)**—China, Yunnan Province, Puer, Jingdong County, Wuliangshan, fallen angiosperm branch, 6 January 2019, C.L. Zhao, CLZhao 9627; 7 January 2019, CLZhao 9831 (SWFC); Wuliangshan, Huangcaoling, fallen angiosperm branch, 5 October 2017, C.L. Zhao, CLZhao 4143 (SWFC); Xujaiba, Aiaoshan Ecological Station, fallen angiosperm branch, 23 August 2018, C.L. Zhao, CLZhao 8413; 24 August 2018, CLZhao 8498, CLZhao 8645; dead tree of angiosperm, 24 August 2018, C.L. Zhao, CLZhao 8620 (SWFC); Zhenyuan County, Ailaoshan, fallen branch of angiosperm, 14 January 2018, C.L. Zhao, CLZhao 5614, CLZhao 5623, CLZhao 5628, CLZhao 5680; 15 January 2018, CLZhao 5821, CLZhao 5841; on the angiosperm stump, 15 January 2018, C.L. Zhao, CLZhao 5873 (SWFC); Damoshan, on the angiosperm trunk, 16 January 2018, C.L. Zhao, CLZhao 6094 (SWFC); Wenshan, Pingba Town, Huguangqing, fallen angiosperm branch, 28 July, 2019, C.L. Zhao, CLZhao 16943, CLZhao 16954, CLZhao 16965, CLZhao 16974, CLZhao 16995 CLZhao 17041, CLZhao 17043, CLZhao 17151; on the angiosperm trunk, 28 July 2019, C.L. Zhao, CLZhao 17023 (SWFC); Pingba National Nature Reserve, allen angiosperm branch, 28 July 2019, C.L. Zhao, CLZhao 17181, CLZhao 17186, CLZhao 17187, CLZhao 17204, CLZhao 17206, CLZhao 17220, CLZhao 17265, CLZhao 17276, CLZhao 17316; on the angiosperm trunk, 3 August 2019, C.L. Zhao, CLZhao 18307; on the stump of *Picea*, 28 July 2019, C.L. Zhao, CLZhao 17226 (SWFC); Xiajie Village, fallen angiosperm branch, 26 July 2019, C.L. Zhao, CLZhao 16278 (SWFC); Xichou County, Dongma Town, Xinzhai Village, fallen angiosperm branch, 16 January 2019, C.L. Zhao, CLZhao 11290; on the angiosperm trunk, 16 January 2019, C.L. Zhao, CLZhao 11305 (SWFC); Lianhuatang Town, Xiangpingshan and Jiguanshan, on the angiosperm trunk, 22 July 2019, C.L. Zhao, CLZhao 15894 (SWFC); Yuxi, Xinping County, Mopanshan National Forest Park, on the fallen branch of *Rhododendron simsii*, 16 January 2017, C.L. Zhao, CLZhao 851 (SWFC); Tea Horse Ancient Road Scenic Spot, fallen branch of angiosperm, 13 January 2018, C.L. Zhao, CLZhao 5361 (SWFC). 

**Notes**—*Crustodontia rhododendri* is sister to *C. chrysocreas* (Berk. & M.A. Curtis) Hjortstam & Ryvarden and then grouped with *C. tongxiniana* (C.L. Zhao) C.C. Chen & Sheng H. Wu (Figure 2), but morphologically *C. chrysocreas* differs in its pruinose hymenophore with the greyish ochraceous hymenial surface covering orange tint, capitate cystidia with pale brownish or yellow encrustations, and slightly thick-walled basidiospores [87]; *C. tongxiniana* differs in its smooth hymenophore with a buff to cinnamon-buff hymenial surface, and wider basidiospores measuring 4.5–5.5 × 2.8–3.5 µm [31].

***Hydnophlebia*** Parmasto, Izv. Akad. Nauk Estonsk. SSR, Ser. Biol. 16: 384. 1967.

**Type species**—*Hydnophlebia chrysorhiza* (Eaton) Parmasto, Eesti NSV Tead. Akad. Toim., Biol. seer 16(4): 384. 1967.

Basidiomata is annual, resupinate, and membranous. Hymenophore reddish-orange, poroid or odontioid-hydnoid, margin with rhizomorphs or fibrillose tissue. The hyphal system is monomitic with simple-septate or clamped generative hyphae. Basidiospores are ellipsoid, colorless, thin-walled, smooth, KI–, CB– [88].

**Figure 5:**
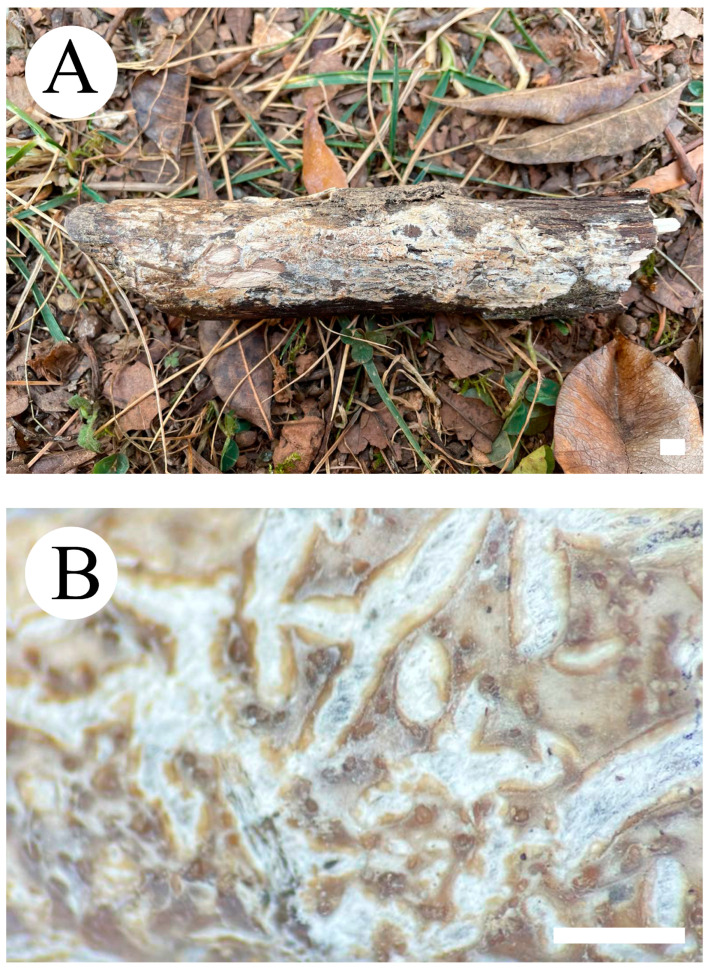
Basidioma of *Hydnophlebia fissurata* (CLZhao 2900, holotype). Bars: (**A**) = 1 cm, (**B**) = 1 mm.

**Figure 6:**
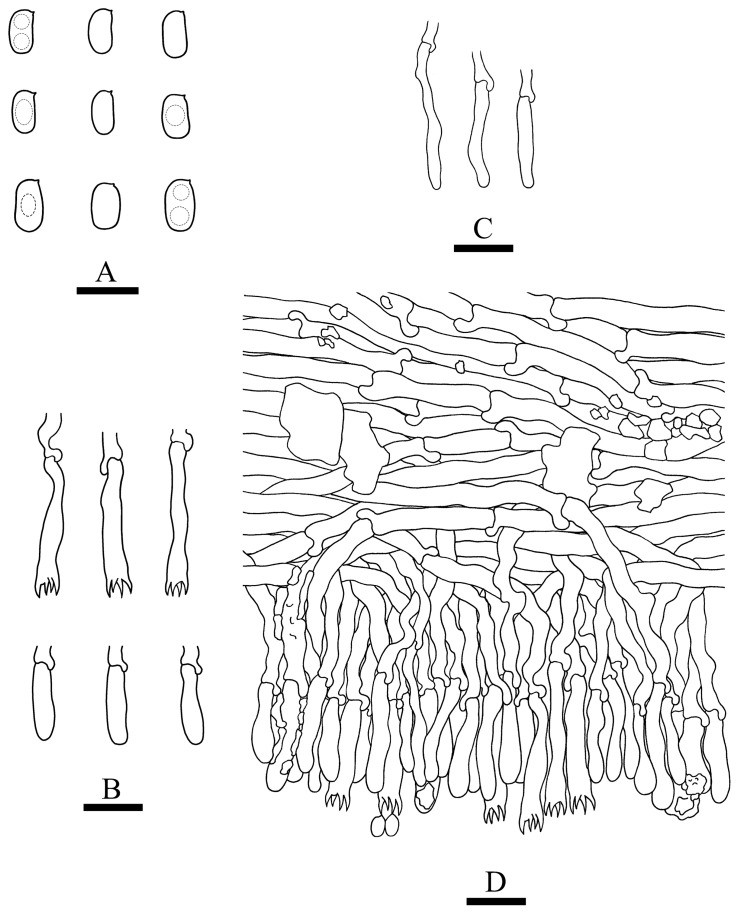
Microscopic structures of *Hydnophlebia fissurata* (drawn from CLZhao 2900, holotype). (**A**) Basidiospores. (**B**) Basidia and basidioles. (**C**) Cystidioles. (**D**) A section of hymenium. Bars: (**A**) = 5 μm, (**B**–**D**) = 10 μm.

***Hydnophlebia fissurata*** C.L. Zhao, sp. nov. (Figure 5 and Figure 6).

MycoBank: MB 843313.

Index Fungorum number: IF843313; Facesoffungi number: FoF12683.

Diagnosis: It differs from *H. fimbriata* by ceraceous basidiomata with grandinoid hymenophore, simple-septate generative hyphae, and ellipsoid, shorter basidiospores as 3–3.8 × 1.6–2.3 µm.

**Holotype**—China, Yunnan Province, Kunming, Xishan District, Haikou Forestry Park, E 103°03′, N 25°37′, alt. 2150 m, on the fallen branch of angiosperm, 16 September 2017, C.L. Zhao, CLZhao 2900 (SWFC).

**Etymology**—Referring to the cracking hymenophore.

Basidiomata annual, resupinate, ceraceous, without odor or taste when fresh, becoming hard upon drying, up to 9 cm long, 2 cm wide, 300–600 µm thick. Hymenophore grandinoid, cream to buff when fresh, buff to pale brown upon drying, cracking. The sterile margin is narrow, cream, and minutely fibrillose. Hyphal structure monomitic; generative hyphae simple-septate, colorless, thin-walled, IKI–, CB–; tissues unchanged in KOH. Subicular hyphae unbranched, 4.5–6.5 μm in diameter; the subhymenial hyphae is unbranched, 2–4 μm in diameter; the presence of numerous yellow to yellowish brown gelatinous substances among generative hyphae. Hymenium cystidia absent; cystidioles colorless, thin-walled, 13.6–28 × 1.3–3.6 µm; basidia cylindrical, with four sterigmata and a basal simple-septate, 21–35 × 2.8–5 µm. Basidiospores are ellipsoid, colorless, thin-walled, and smooth, often with 1–2 oil drops, IKI–, CB–, (2.8–)3–3.8 × 1.6–2.3 µm, L = 3.23 µm, W = 1.93 µm, Q = 1.72 (n = 30/1).

**Distribution and ecology**—The species is known from Yunnan Province, China, in a subtropical evergreen broad-leaved forest. It grows on moderately decayed angiosperm wood and causes a white rot.

**Notes**—*Hydnophlebia fissurata* groups with *Phlebia acanthocystis* Gilb. & Nakasone and *P. caspica* Hallenb.; however, morphologically, *P. acanthocystis* differs in its odontoid to hydnoid hymenophore with cream to pale brown hymenial surface, obclavate cystidia, and broadly elhipsoid basidiospores [89]; *P. caspica* differs in the crustaceous basidiomata, with both larger cystidia (40–67 × 4–4.5 µm) and basidiospores (4–5 × 2–2.5 µm) [90].

***Hydnophlebia acanthocystis*** (Gilb. & Nakasone) C.L. Zhao, comb. nov.

MycoBank: MB 843314.

Index Fungorum number: IF843314; Facesoffungi number: FoF12680.

**Basionym**—*Phlebia acanthocystis* Gilb. & Nakasone, in Nakasone & Gilbertson, Folia cryptog. Estonica 33: 85. 1998.

**Notes**—Morphologically, this species is characterized by the odontoid to hydnoid hymenial surface with rhizomorphs, which accords with the character of the genus *Hydnophlebia*. Phylogenetically, it nests into *Hydnophlebia*, suggesting a new combination in the current study (Figure 2).

***Hydnophlebia caspica*** (Hallenb.) C.L. Zhao, comb. nov.

MycoBank: MB 843315.

Index Fungorum number: IF843315; Facesoffungi number: FoF12681.

**Basionym**—*Phlebia caspica* Hallenb., Mycotaxon 11(2): 460. 1980.

**Notes**—This species has a minutely fibrillose hymenophore, and it clusters into the genus Hydnophlebia based on the present molecular study (Figure 2).

***Hydnophlebia fimbriata*** (C.L. Zhao & Y.C. Dai) C.L. Zhao, comb. nov.

MycoBank: MB 843316.

Index Fungorum number: IF843316; Facesoffungi number: FoF12682.

**Basionym**—*Ceriporiopsis fimbriata* C.L. Zhao & Y.C. Dai, in Zhao, Wu, Liu & Dai, Nova Hedwigia 101(3–4): 409. 2015.

**Notes**—It has a poroid hymenophore with a fimbriate margin in morphology, and it nests into the genus *Hydnophlebia* on the basis of the phylogeny (Figure 2).

***Luteoporia*** F. Wu, Jia J. Chen & S.H. He, in Wu, Yuan, Chen & He, Phytotaxa 263(1): 37. 2016.

**Type species**—*Luteoporia albomarginata* F. Wu, Jia J. Chen & S.H. He, in Wu, Yuan, Chen & He, Phytotaxa 263(1): 37. 2016.

Basidiomata resupinate, ceraceous. Hymenophore is poroid or odontioid to hydnoid with a pale yellow to golden yellow hymenial surface, tissue becoming reddish or purple in KOH. The hyphal system is monomitic, having generative hyphae with clamp connections, usually with swollen tips. Cystidia-like hyphae projecting out of hymenium, and cystidioles aree present. Basidia subclavate to barrel-shaped, bearing four sterigmata and a basal clamp connection. Basidiospores are oblong–ellipsoid to ellipsoid, colorless, thin-walled, and smooth, KI–, CB– [62]. 

**Figure 7:**
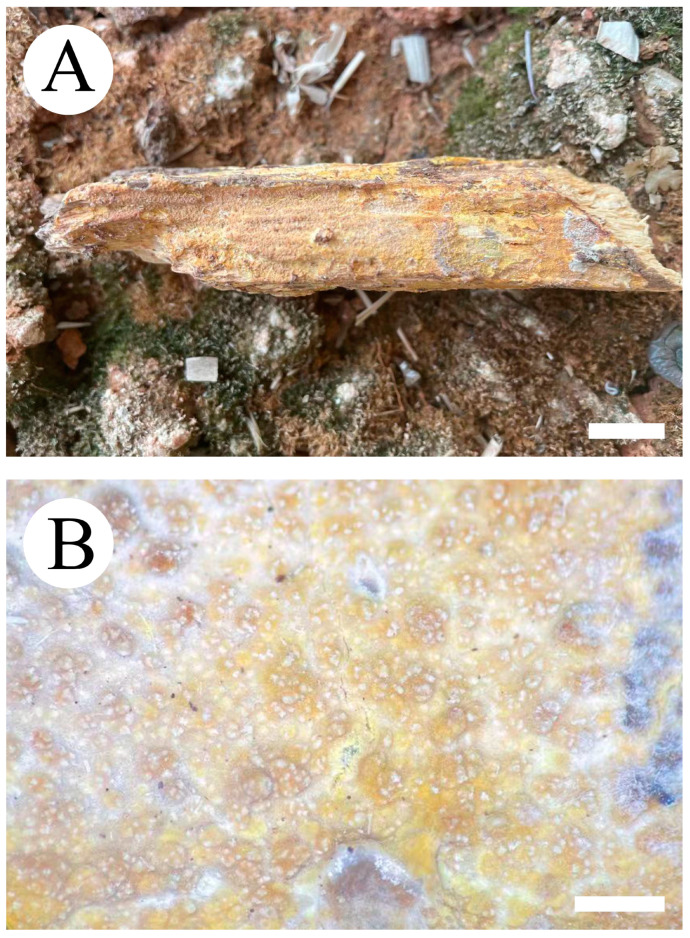
Basidioma of *Luteoporia straminea* (CLZhao 18947, holotype). Bars: (**A**) = 1 cm, (**B**) = 1 mm.

**Figure 8:**
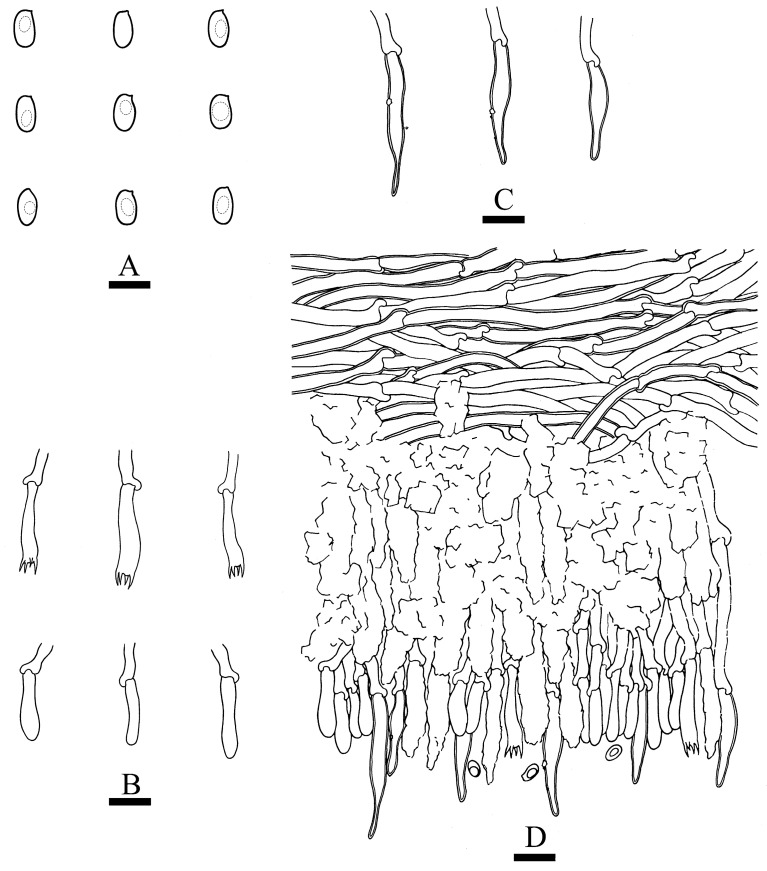
Microscopic structures of *Luteoporia straminea* (drawn from CLZhao 18947, holotype). (**A**) Basidiospores. (**B**) Basidia and basidioles. (**C**) Cystidia. (**D**) A section of hymenium. Bars: (A) = 5 μm, (**B**–**D**) = 10 μm.

***Luteoporia straminea*** C.L. Zhao, sp. nov. (Figure 7 and Figure 8).

MycoBank: MB 843318.

Index Fungorum number: IF843318; Facesoffungi number: FoF12684.

Diagnosis: It is characterized by annual basidiomata, the odontioid hymenophore has a straw to pale orange color, a monomitic hyphal structure with clamped generative hyphae, subuliform, colorless, thick-walled cystidia occasionally covered with small yellowish crystals.

**Holotype**—China, Yunnan Province, Honghe, Pingbian County, Daweishan National Nature Reserve, E 102°06′, N 22°49′, alt. 1800 m, on the angiosperm trunk, 9 June 2020, C.L. Zhao, CLZhao 18947 (SWFC).

**Etymology**—Referring to the straw color of the hymenial surface.

Basidiomata annual, resupinate, ceraceous, without odor or taste when fresh, becoming hard upon drying, up to 12 cm long, 6.5 cm wide, 200–400 µm thick. Hymenophore odontioid, buff to slightly straw when fresh, straw to pale orange upon drying, tissue becoming reddish in KOH. The sterile margin is narrow and slightly straw. The hyphal structure is monomitic; generative hyphae are clamped, colorless, thin- to thick-walled, and IKI–, CB–; tissues unchanged in KOH. Subicular hyphae unbranched, 3–5 μm in diameter; subhymenial hyphae unbranched, 2–4 μm in diameter; the presence of numerous yellow to yellowish brown gelatinous substances below subhymenium. Hymenium cystidia subuliform, colorless, thick-walled, occasionally covering with small yellowish crystals, 24.1–37.9 × 4.1–5.3 μm, cystidioles are absent; basidia clavate, with four sterigmata and a basal clamp connection, 17.5–24.1 × 2.7–4.2 μm. Basidiospores ellipsoid, colorless, thin-walled, smooth, often with 1 oil drop, IKI–, CB–, (3.7–)4–4.6(–4.8) × (2–)2.2–3(–3.2) μm, L = 4.24 µm, W = 2.55 µm, Q = 1.57–1.69 (n = 90/3).

**Distribution and ecology**—The species is known from Yunnan Province, China, in a subtropical evergreen broad-leaved forest. It grows on moderately decayed angiosperm wood and causes a white rot.

**Specimens examined (paratypes)**—China, Yunnan Province, Honghe, Xichou County, Jiguanshan Forestry Park, on the angiosperm trunk, 22 July 2020, C.L. Zhao, CLZhao 15724, CLZhao 15749 (SWFC); Puer, Zhenyuan County, Heping Town, Liangzi Village, Ailaoshan, on the stump of angiosperm, 15 January 2018, C.L. Zhao, CLZhao 5794 (SWFC).

**Notes**—*Luteoporia straminea* is sister to *L. lutea* (G. Cunn.) C.C. Chen & Sheng H. Wu (Figure 2), but the latter differs in its golden yellow basidiomata, presence of slightly thick-walled fusoid cystidioles, and slightly thick-walled ellipsoid basidiospores [34].

***Merulius*** Fr., Syst. mycol. (Lundae) 1: 326. 1821.

**Type species**—*Merulius tremellosus* Schrad., Spicil. fl. germ. 1: 139. 1794.

Basidiomata is resupinate to effused–reflexed, ceraceous to gelatinous. The hymenopis hore is surface mainly merulioid, sometimes poroid to grandinoid. The hyphal system monomitic; generative hyphae with clamp connections. Cystidia and cystidioles are present or absent. Basidiospores allantoid or ellipsoid, colorless, thin-walled, and smooth, IKI–, CB– [10].

**Figure 9:**
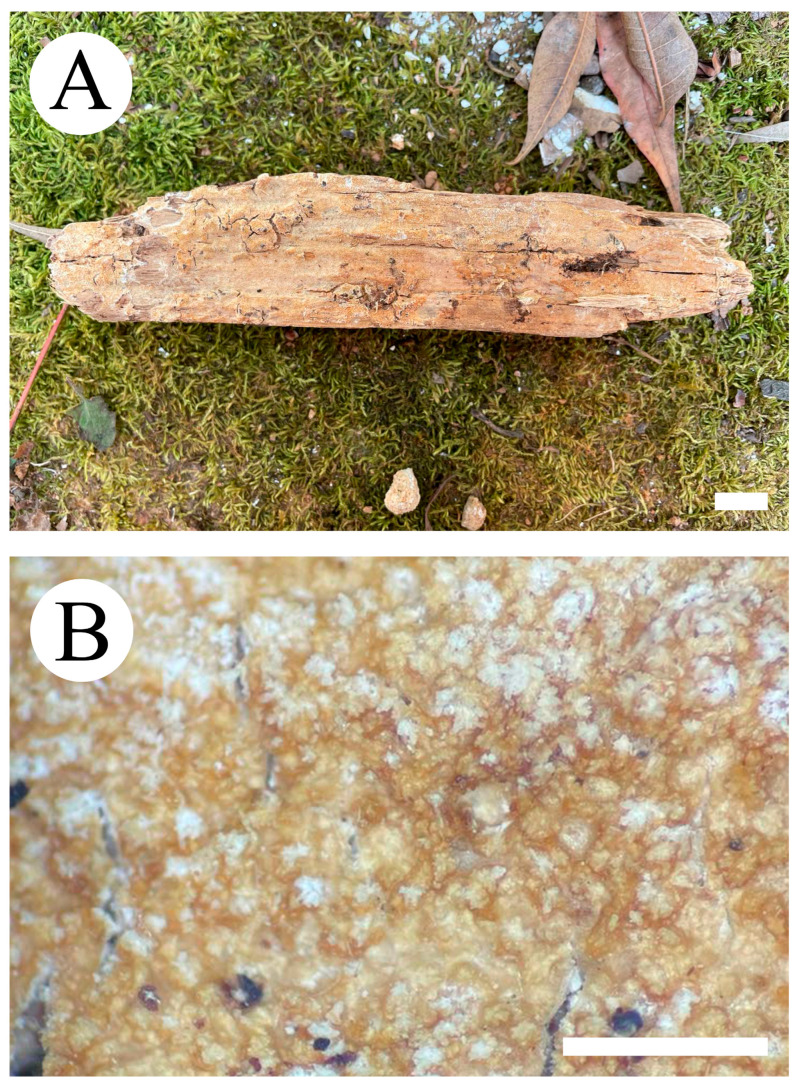
Basidioma of *Merulius sinensis* (CLZhao 2562, holotype). Bars: (**A**) = 1 cm, (**B**) = 1 mm.

**Figure 10:**
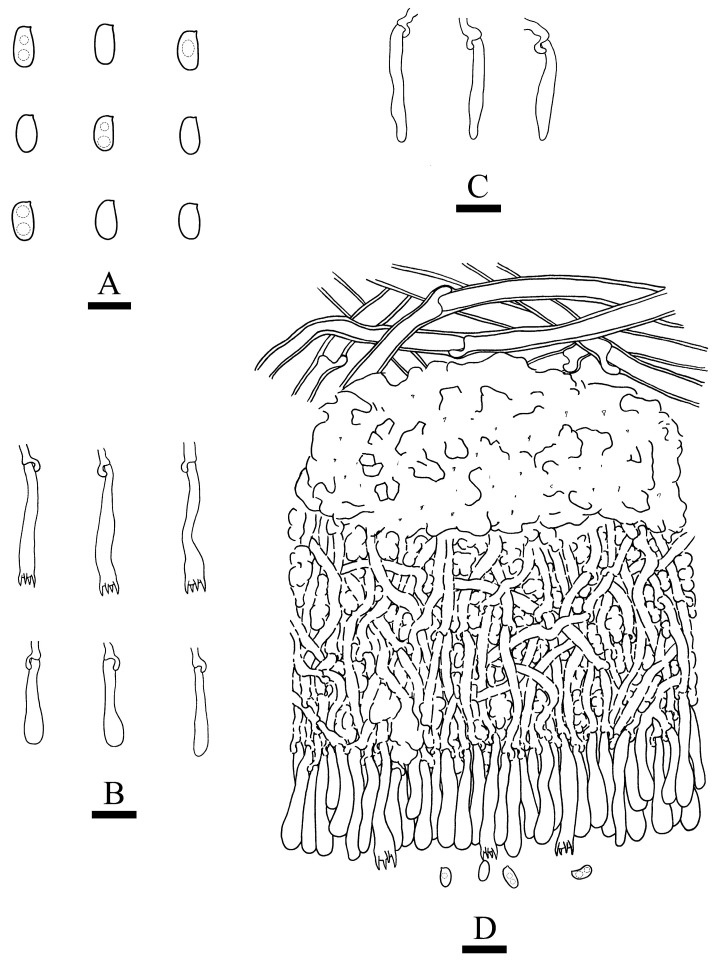
Microscopic structures of *Merulius sinensis* (drawn from CLZhao 2562, holotype). (**A**) Basidiospores. (**B**) Basidia and basidioles. (**C**) Cystidioles. (**D**) A section of hymenium. Bars: (**A**) = 5 μm, (**B**–**D**) = 10 μm.

***Merulius sinensis*** C.L. Zhao, sp. nov. (Figure 9 and Figure 10).

MycoBank: MB 843319.

Index Fungorum number: IF843319; Facesoffungi number: FoF12687.

Diagnosis: It is characterized by annual basidiomata with a grandinoid hymenophore, the presence of numerous larger golden gelatinous substances below the subhymenium and ellipsoid, colorless, thin-walled, with a smooth basidiospores measuring 3.8–4.5 × 2–2.6 μm and it grows on moderately decayed angiosperm wood in a subtropical evergreen broad-leaved forest and causes a white rot.

**Holotype**—China, Yunnan Province, Yuxi, Xinping County, Mopanshan National Forestry Park, E 102°48′, N 24°51′, alt. 1980 m, on the fallen branch of angiosperm, 20 August 2017, C.L. Zhao, CLZhao 2562 (SWFC).

**Etymology**—Referring to the provenance (China) of the type specimen.

Basidiomata annual, resupinate, ceraceous, without odor or taste, when fresh, becoming coriaceous upon drying, up to 13 cm long, 3.5 cm wide, 100–300 µm thick. Hymenophore grandinoid, pinkish buff when fresh, peach upon drying. The sterile margin is narrow, buff to peach. The hyphal structure is monomitic; generative hyphae clamped, colorless, thin- to thick-walled, IKI–, CB–; tissues unchanged in KOH. Subicular hyphae thick-walled, unbranched, 4–5.5 μm in diameter; subhymenial hyphae unbranched, 1.5–3.5 μm in diameter; the presence of numerous larger golden gelatinous substances below subhymenium. Hymenium cystidia absent; cystidioles colorless, thin-walled, smooth, 18.7–26.4 × 2.3–3.5 µm; basidia cylindrical, with four sterigmata and a basal clamp connection, 19.7–29.2 × 2.9–4.5 µm. Basidiospores ellipsoid, colorless, thin-walled, smooth, often with 1–2 oil drops, IKI–, CB–, (3.6–)3.8–4.5(–4.7) × (1.8–)2–2.6 μm, L = 4.13 µm, W = 2.24 µm, Q = 1.85 (n = 30/1).

**Distribution and ecology**—The species is known from Yunnan Province, China, in a subtropical evergreen broad-leaved forest. It grows on moderately decayed angiosperm wood and causes a white rot.

**Notes**—This species groups with *Phlebia nantahaliensis* Nakasone & Burds. (Figure 2), but the latter distinguishes from *Merulius sinensis* due to its very thin basidiomata and allantoid, narrower basidomata (4.5–4.5 × 1.8–2 μm) [11].

***Merulius fuscotuberculatus*** (C.L. Zhao) C.L. Zhao, comb. nov.

MycoBank: MB 843320.

Index Fungorum number: IF843320; Facesoffungi number: FoF12685.

**Basionym**—*Phlebia fuscotuberculata* C.L. Zhao, in Huang & Zhao, Mycol. Progr. 19: 761. 2020.

**Notes**—This species was located in *Phlebia* s.l. (31), but the genus *Merulius* shows as a single lineage inferred from the phylogenetical data in the present study (Figure 2); therefore, we propose a new combination species, *Merulius fuscotuberculata*.

***Meruliusnantahaliensis*** (Nakasone & Burds.) C.L. Zhao, comb. nov.

MycoBank: MB 843321.

Index Fungorum number: IF843321; Facesoffungi number: FoF12686.

**Basionym**—*Phlebia nantahaliensis* Nakasone & Burds., Mycotaxon 54: 348 (1995).

***Merulius tomentopileatus*** (C.L. Zhao) C.L. Zhao, comb. nov.

MycoBank: MB 843322.

Index Fungorum number: IF843322; Facesoffungi number: FoF12688.

**Basionym**—*Phlebia tomentopileata* C.L. Zhao, in Huang & Zhao, Mycol. Progr. 19: 762. 2020.

**Notes**—This species has the typical merulioid hymenophore, which is consistent with the representative morphological character of the genus *Merulius*, and phylogenetically it clusters into the genus *Merulius* (Figure 2); therefore, we proposed a new combination, *Merulius tomentopileatus*.

***Mycoacia*** Donk, Medded. Nedl. Mycol. Ver. 18-20: 150. 1931.

**Type species**—*Mycoacia fuscoatra* (Fr.) Donk, Medded. Nedl. Mycol. Ver. 18-20: 152. 1931.

Basidiomata resupinate, adnate, effused, and ceraceous. Hymenophore odontioid to hydnoid, aculei conical or cylindrical. *Hyphal system* monomitic, generative hyphae with clamp connections (nodose septate), IKI–, CB–; tissues unchanged in KOH. Cystidia (leptocystidia) often present; basidia narrowly clavate, with a basal clamp connection, producing four sterigmata. Basidiospores narrowly ellipsoid, cylindrical or allantoid, colorless, thin-walled, smooth, IKI–, CB– [91].

***Mycoacia tuberculata*** (Berk. & M.A. Curtis) C.L. Zhao, comb. nov.

MycoBank: MB 843323.

Index Fungorum number: IF843323; Facesoffungi number: FoF12689.

**Basionym**—*Grandinia tuberculata* Berk. & M.A. Curtis, Hooker’s J. Bot. Kew Gard. Misc. 1: 237. 1849.

***Mycoaciella*** J. Erikss. & Ryvarden, in Eriksson, Hjortstam & Ryvarden, Cortic. N. Eur. (Oslo) 5: 901. 1978.

**Type species**—*Mycoaciella bispora* (Stalpers) J. Erikss. & Ryvarden, in Eriksson, Hjortstam & Ryvarden, Cortic. N. Eur. (Oslo) 5: 902. 1978.

Basidiomata resupinate, effused, ceraceous. Hymenophore hydnoid to grandinoid or tuberculata. The hyphal system is monomitic to dimitic, having generative hyphae with simple septa or clamp connections, and the skeletal hyphae are thick-walled. Cystidia is cylindrical, thin-walled, with an apical globule of excreted, resinous matter. *Basidia* clavate, with four sterigmata. Basidiospores are narrowly ellipsoid, thin-walled, and smooth, IKI–, CB– [92].

**Figure 11:**
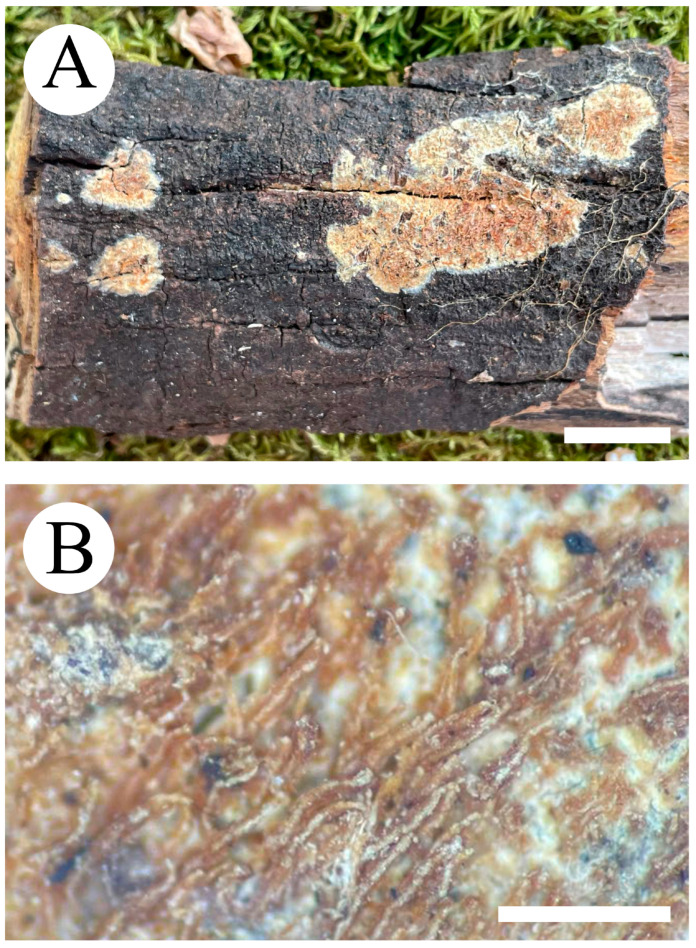
Basidioma of *Mycoaciella brunneospina* (CLZhao 15876, holotype). Bars: (**A**) = 1 cm, (**B**) = 1 mm.

**Figure 12:**
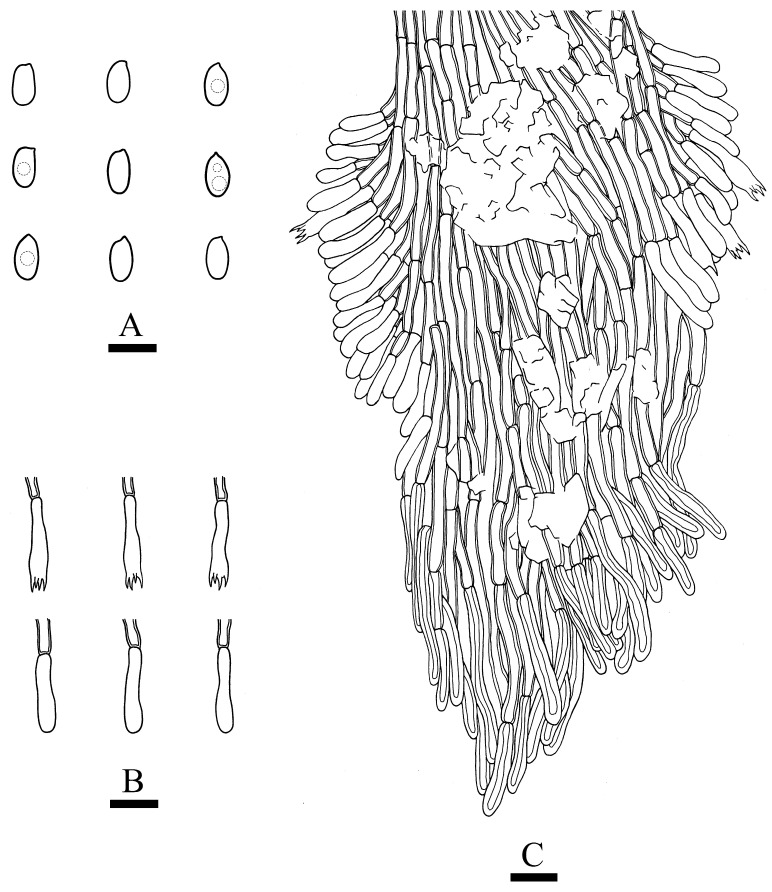
Microscopic structures of *Mycoaciella brunneospina* (drawn from CLZhao 15876, holotype). (**A**) Basidiospores. (**B**) Basidia and basidioles. (**C**) A section of hymenium. Bars: (**A**) = 5 μm, (**B**,**C**) = 10 μm.

***Mycoaciella brunneospina*** C.L. Zhao, sp. nov. (Figure 11 and Figure 12).

MycoBank: MB 843324.

Index Fungorum number: IF843324; Facesoffungi number: FoF12690.

Diagnosis: It is characterized by annual basidiomata with a hydnoid hymenophore with cylindrical spines of 4–5/mm. It is slightly brown to brown, having a monomitic hyphal structure with simple septate generative hyphae and ellipsoid, colorless, thin-walled, and smooth basidiospores.

**Holotype**—China, Yunnan Province, Wenshan, Xichou County, Jiguanshan Forestry Park, E 104°39′, N 23°10′, alt. 1800 m, on the angiosperm trunk, 22 July 2019, C.L. Zhao, CLZhao 15876 (SWFC).

**Etymology**—Referring to the brown spine of the type specimen.

Basidiomata annual, resupinate, ceraceous, without odor or taste when fresh, becoming coriaceous upon drying, up to 8 cm long, 2.5 cm wide, 0.8–1.5 mm thick. Hymenophore is hydnoid, having cylindrical spines 4–5/mm. It is buff to slightly brown when fresh, slightly brown to brown upon drying. The sterile margin is narrow, buff, fimbriate. The hyphal structure is monomitic; generative hyphae with simple septa, unbranched, 3–5 μm in diameter, colorless, thick-walled, IKI–, CB–; tissues unchanged in KOH. Hymenium cystidia and cystidioles are absent; the presence of larger, yellow to yellowish brown gelatinous substance; basidia clavate, with four sterigmata and a simple basal septum, 11.5–19.5 × 4–5 μm. Basidiospores ellipsoid, colorless, thin-walled, smooth, often with 1–2 oil drops, IKI–, CB–, (3.7–)3.9–4.8 × (1.8–)2–2.7 μm, L = 4.17 µm, W = 2.36 µm, Q = 1.77 (n = 30/1).

**Distribution and ecology**—The species is known from Yunnan Province, China, in a subtropical evergreen broad-leaved forest. It grows on moderately decayed angiosperm wood and causes a white rot.

**Notes**—*Mycoaciella brunneospina* groups with *M. bispora* (Stalpers) J. Erikss. & Ryvarden in the phylogenetic tree (Figure 2), but *M. bispora* differs in its dimitic hyphal system and larger basidiospores (5–6.5 × 2.5–3 μm) [93]. *Mycoaciella efibulata* C.C. Chen & Sheng H. Wu differs in its forming small patches of basidiomata with a yellowish-brown or grayish-brown hymenial surface and dextrinoid skeletal hyphae [34]. *Mycoaciella badia* (Pat.) Hjortstam & Ryvarden differs from *M*. *brunneospina* by the presence of resinous-capped cystidia [14]. *Mycoacia aurea* (Fr.) J. Erikss. & Ryvarden differs in its membranaceous basidiomata with a cream to yellowish hymenophore, fibrillose margin, and allantoid basidiospores [7].

***Mycoaciella uda*** (Fr.) C.L. Zhao, comb. nov.

MycoBank: MB 843325.

Index Fungorum number: IF843325; Facesoffungi number: FoF12691.

**Basionym**—*Hydnum udum* Fr., Syst. mycol. (Lundae) 1: 422. 1821.

**Notes**—It is characterized by the odontioid hymenophore with aculei up to 1–2 mm long, at first, light yellowish, more or less ochraceous hymenial surface when mature, margin more or less fibrillose, and a monomitic hyphal system with clamped generative hyphae encrusted with several crystals in the aculei, more or less fusoid, slightly projecting cystidiols and narrowly ellipsoid basidiospores [13]. We propose it as a new combination mainly based on the current molecular result (Figure 2).

***Phlebia*** Fr., Syst. mycol. (Lundae) 1: 426. 1821.

**Type species**—*Phlebia radiata* Fr., Syst. mycol. (Lundae) 1: 427. 1821.

Basidiomata effused or partly pileate basidiocarps with a subceraceous to subgelatinous consistency when fresh and membranaceous to coriaceous when dry. The hymenophore is smooth, tuberculata, phlebioid, odontioid, merulioid, or poroid. The hyphal structure is monomitic, having generative hyphae with clamp connections; basidia narrowly clavate. Basidiospores are colorless, thin-walled, smooth, allantoid to ellipsoid, acyanophilous, in-amyloid, and non-dextrinoid [7,8,31,34].

**Figure 13:**
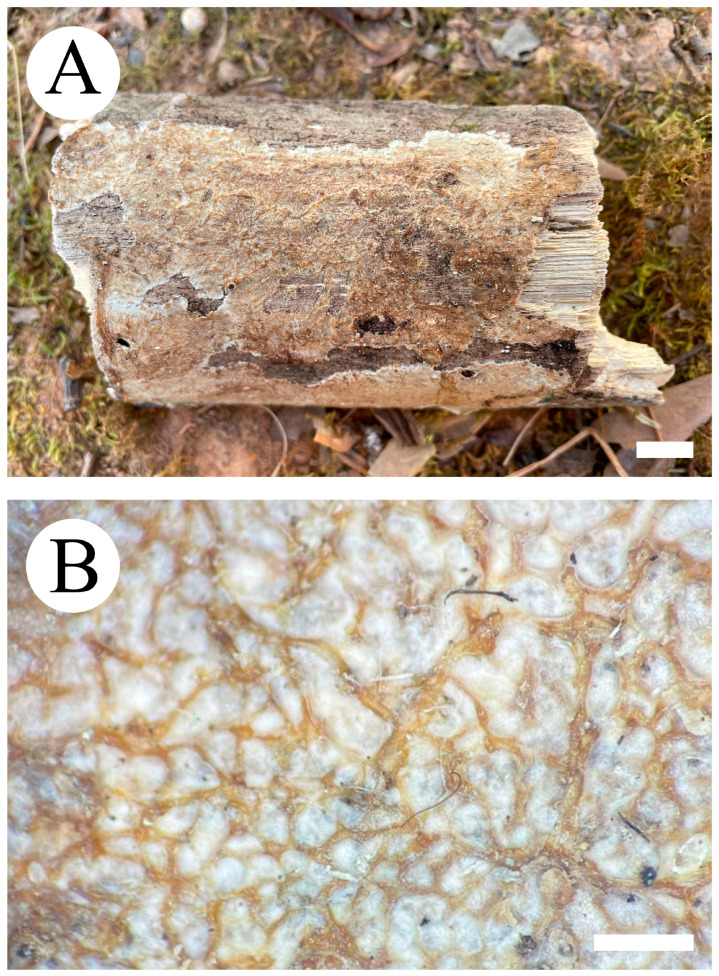
Basidioma of *Phlebia niveomarginata* (CLZhao 19089, holotype). Bars: (**A**) = 1 cm, (**B**) = 1 mm.

**Figure 14:**
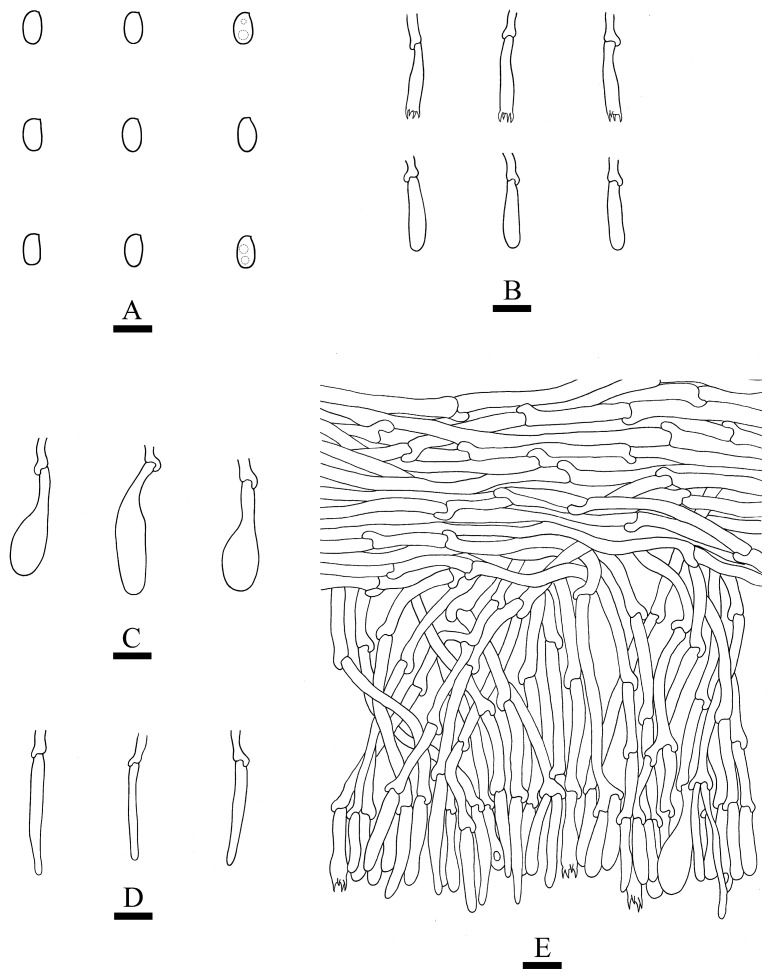
Microscopic structures of *Phlebia niveomarginata* (drawn from CLZhao 19089, holotype). (**A**) Basidiospores. (**B**) Basidia and basidioles. (**C**) Cystidia. (**D**) Cystidioles. (**E**) A section of hymenium. Bars: (**A**) = 5 μm, (**B**–**E**) = 10 μm.

***Phlebia niveomarginata*** C.L. Zhao, sp. nov. (Figure 13 and Figure 14).

MycoBank: MB 843326.

Index Fungorum number: IF843326; Facesoffungi number: FoF12692.

Diagnosis: It is characterized by annual basidiomata with a phlebioid hymenophore, having a greyish-brown to brown hymenial surface, a monomitic hyphal structure having generative hyphae clamped at all primary septa, and ellipsoid, colorless, thin-walled, and smooth, IKI–, CB– basidiospores.

**Holotype**—China, Yunnan Province, Honghe, Pingbian County, Daweishan National Nature Reserve, E 102°06′, N 22°49′, alt. 1800 m, on a fallen branch of angiosperm, 9 Jun 2020, C.L. Zhao, CLZhao 19089 (SWFC).

**Etymology**—Refers to the white margin of type specimens.

Basidiomata annual, resupinate, ceraceous to subgelatinous, without odor or taste, when fresh, becoming coriaceous upon drying, up to 15 cm long, 6 cm wide, 300–500 µm thick. Hymenophore phlebioid, hymenial surface cream to greyish brown when fresh, greyish brown to brown upon drying. The sterile margin is narrow, 1–2 mm wide, and white. The hyphal structure is monomitic; generative hyphae clamped at all primary septa, colorless, thin-walled, IKI–, CB–; tissues unchanged in KOH. Subicular hyphae unbranched, 3.5–6 μm in diameter; subhymenial hyphae infrequently branched, 2–4.5 μm in diameter. Hymenium cystidia pear-shaped, thin-walled, smooth, 25–29.5 × 7.5–10 μm; cylindrical paraphysoid hyphae present, colorless, and thin-walled, 20–29 × 2–4 µm; basidia cylindrical, with four sterigmata and a basal clamp connection, 16–22.5 × 2.5–4.5 µm. Basidiospores ellipsoid, colorless, thin-walled, smooth, IKI–, CB–, 3.7–4.7(–4.9) × (1.7–)1.8–2.5(–2.6) μm, L = 4.12 µm, W = 2.22 µm, Q = 1.81–1.88 (n = 60/2).

**Distribution and ecology**—The species is known from Yunnan Province of China in a temperate forest area. It grows on small-sized and broad-leaved forest trees and provokes white rot. 

**Specimen examined (paratype)**—China, Yunnan Province, Honghe, Pingbian County, Daweishan National Nature Reserve, E 102°06′, N 22°49′, alt. 1800 m, on an angiosperm trunk, 9 June 2020, C.L. Zhao, CLZhao 18972 (SWFC).

**Notes**—*Phlebia niveomarginata* clusters into *Phlebia* s.s., and it forms a single lineage, so we proposed it as a new species. Morphologically, *Phlebia centrifuga* P. Karst. differs in its fibrillose margin, encrusted subhymenium and arranged in a differenciate layer, larger basidiospores (6–9 × 2.5–3 μm) [7]. The species *P. radiata* differs from *P. niveomarginata* by richly branched generative hyphae, the gelatinous matrix embedded among hyphae, long tubular to more or less long clavate cystidia, and allantoid basidiospores [7]. *Phlebia rufa* (Pers.) M.P. Christ. differs in its pale yellowish, reddish, or brownish hymenial surface, larger cystida (40–100 × 6–15 μm) and suballantoid basdiospores (4.5–6.5 × 2–2.5 μm) [7].

**Figure 15:**
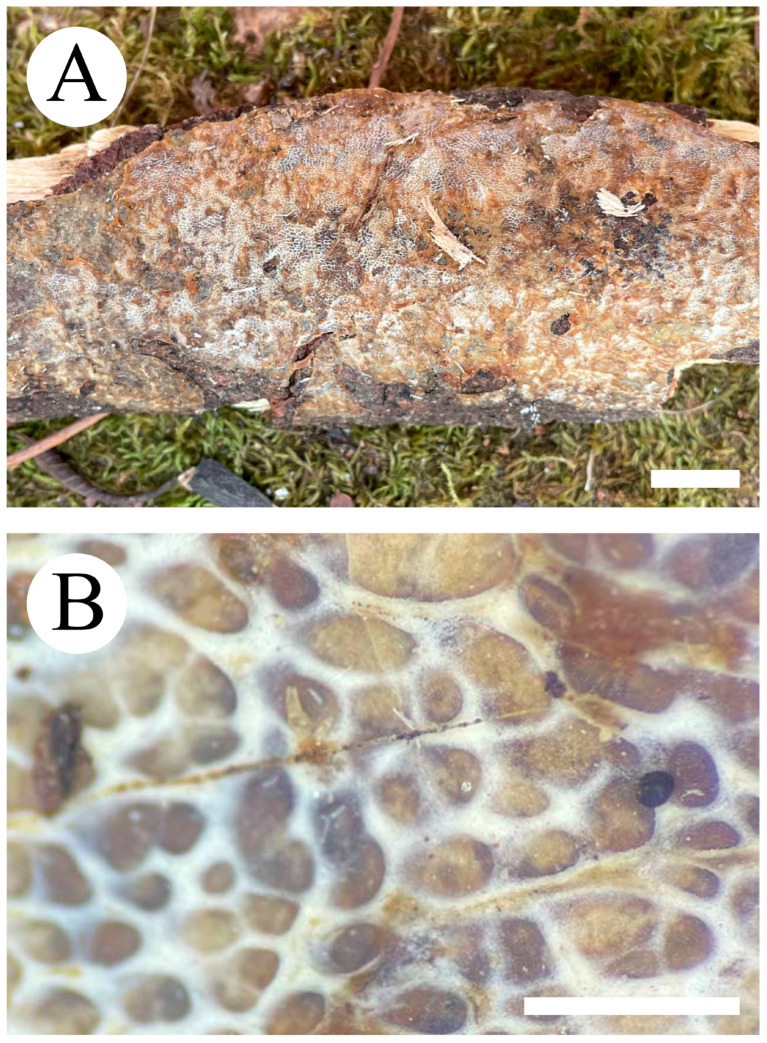
Basidioma of *Phlebia poroides* (CLZhao 16121, holotype). Bars: (**A**) = 1 cm, (**B**) = 1 mm.

**Figure 16:**
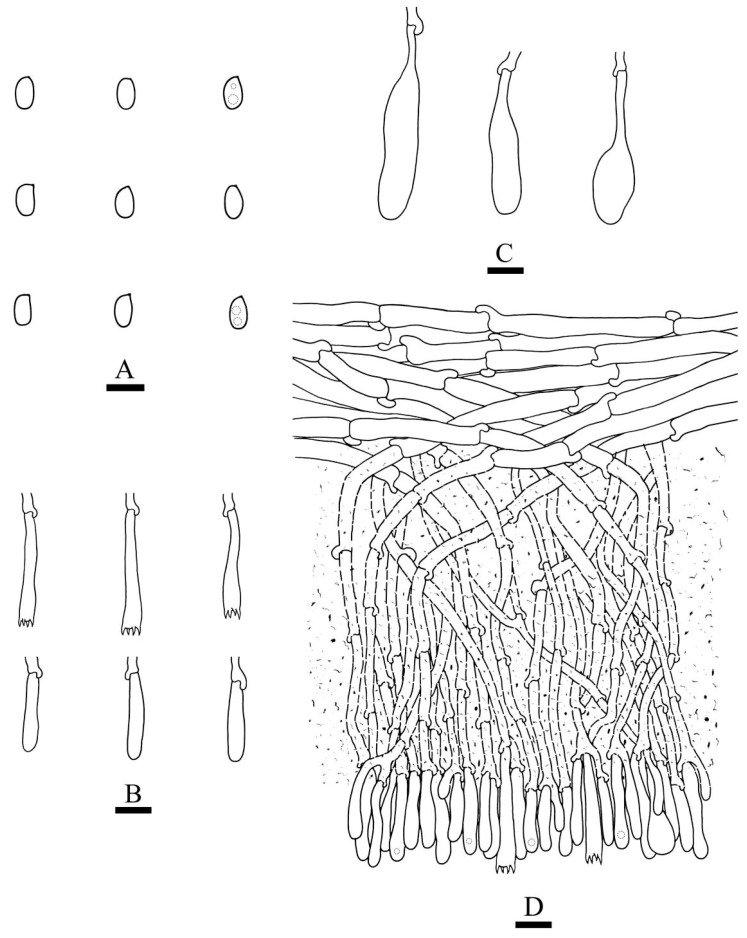
Microscopic structures of *Phlebia poroides* (drawn from CLZhao 16121, holotype). (**A**) Basidiospores. (**B**) Basidia and basidioles. (**C**) Cystidia. (**D**) A section of hymenium. Bars: (**A**) = 5 μm, (**B**–**D**) = 10 μm.

***Phlebia poroides*** C.L. Zhao, sp. nov. (Figure 15 and Figure 16).

MycoBank: MB 843327.

Index Fungorum number: IF843327; Facesoffungi number: FoF12693.

Diagnosis: It is characterized by annual, resupinate basidiomata with poroid hymenophore, round, thin-walled, entire pores (3–4/mm), a monomitic hyphal structure having generative hyphae clamp connections, and ellipsoid, colorless, thin-walled, and smooth basidiospores.

**Holotype**—China, Yunnan Province, Wenshan, Pingba Town, Wenshan National Nature Reserve, E 104°31′, N 23°22′, alt. 1720 m, on the fallen branch of angiosperm, 25 July 2019, C.L. Zhao, CLZhao 16121 (SWFC).

**Etymology**—Referring to the poroid hymenophore.

Basidiomata annual, resupinate, ceraceous, without odor or taste, when fresh, becoming hard and fragile upon drying, up to 11 cm long, 5 cm wide, 100–300 µm thick. Hymenophore is poroid, buff when fresh, buff to slightly brown upon drying, and the pores are 3–4/mm, round, thin-walled, and entire. The sterile margin is narrow and slightly brown. The hyphal structure is monomitic; generative hyphae clamp connections, colorless, thin-walled, IKI–, CB–; tissues unchanged in KOH. Subicular hyphae unbranched, 4–6.5 μm in diameter; subhymenial hyphae rarely branched, 2–4.5 μm in diameter; the presence of numerous brown sand-shaped substances among subhymenium. Hymenium cystidia pear-shaped, colorless, thin-walled, and smooth, 21.9–47.3 × 7–10.5 μm, cystidioles are absent; basidia cylindrical, with four sterigmata and a basal clamp connection, 16–28.6 × 3.2–4.9 μm. Basidiospores ellipsoid, colorless, thin-walled, smooth, often with 1–2 oil drops, IKI–, CB–, (3.2–) 3.4–4.2 (–4.5) × 1.7–2.5 (–2.6) μm, L = 3.72 µm, W = 2.03 µm, Q = 1.77–1.83 (n = 60/2).

**Distribution and ecology**—The species is known from Yunnan Province, China, in a subtropical evergreen broad-leaved forest. It grows on moderately decayed angiosperm wood and causes a white rot.

**Specimens examined (paratypes)**—China, Yunnan Province, Honghe, Pingbian County, Daweishan National Nature Reserve, on the fallen branch of angiosperm, 2 August 2019, C.L. Zhao, CLZhao 18421; on the trunk of angiosperm, 6 June 2020, C.L. Zhao, CLZhao 18594 (SWFC).

**Notes**—*Phlebia poroides* is sister to *P. acerina* Peck with lower supports (Figure 2), but *P. acerina* differs in its orange to brown hymenophore and thick-walled subicular generative hyphae and smaller basidiospores (4.7–5.2 × 2–2 μm) [94].

***Phlebicolorata*** C.L. Zhao gen. nov.

MycoBank: MB 843328.

Index Fungorum number: IF843328; Facesoffungi number: FoF12698.

Diagnosis: It is characterized by annual, resupinate basidiomata with a tuberculata or poroid hymenophore having a vivid or bright-colored hymenial surface, a monomitic hyphal system having the generative hyphae with clamp connections and colorless, thin-walled, smooth, and broadly ellipsoid to short cylindrical basidiospores.

**Type species**—*Phlebicolorata brevispora* (Nakasone) C.L. Zhao.

**Etymology**—*Phlebicolorata* (Lat.): referring to the vivid hymenial surface.

Basidiomata annual, resupinate. Hymenophore is tuberculata or poroid; the hymenial surface is vivid or bright-colored. The hyphal system is monomitic, having generative hyphae with clamp connections, IKI–, CB–; tissues becoming vinaceous brown to black in KOH. Basidiospores are colorless, thin-walled, smooth, and broadly ellipsoid to short cylindrical, which are acyanophilous, inamyloid, and non-dextrinoid.

***Phlebicolorataalboaurantia*** (C.L. Zhao, B.K. Cui & Y.C. Dai) C.L. Zhao, comb. nov.

MycoBank MB 843329.

Index Fungorum number: IF843329; Facesoffungi number: FoF12694.

**Basionym**—*Ceriporiopsis alboaurantia* C.L. Zhao, B.K. Cui & Y.C. Dai, in Zhao & Cui, Phytotaxa 164(1): 22. 2014.

**Notes**—This species is characterized by the poroid basidiomata with apricot-orange to a dark orange surface with a reddish tinge and a monomitic hyphal structure having generative hyphae with clamp connections and ellipsoid, colorless, thin-walled, and smooth basidiospores [43]. In the present study, it groups into the genus *Phlebicolorata* based on the phylogenetic tree (Figure 2) and we propose to transfer it to this genus as a new combination.

***Phlebicoloratabrevispora*** (Nakasone) C.L. Zhao, comb. nov.

MycoBank: MB 843330.

Index Fungorum number: IF843330; Facesoffungi number: FoF12695.

**Basionym**—*Phlebia brevispora* Nakasone, in Nakasone & Eslyn, Mycologia 73(5): 805. 1981.

**Notes**—Morphologically, it has tuberculata basidiomata with the light brownish olive hymenophore, a monomitic hyphal system, and ellipsoid to short cylindrical basidiospores [95], which size up the characters of *Phlebicolorata*. Phylogenetically, it nests into the genus *Phlebicolorata* and then groups with *Phlebicolorata rosea* (Figure 2).

***Phlebicolorata pseudoplacenta*** (Vlasák & Ryvarden) C.L. Zhao, comb. nov.

MycoBank MB 843331.

Index Fungorum number: IF843331; Facesoffungi number: FoF12696.

**Basionym**—*Ceriporiopsis pseudoplacenta* Vlasák & Ryvarden, in Vlasák, Vlasák & Ryvarden, Mycotaxon 119: 222. 2012.

**Notes**—It is characterized by a poroid basidiomata having a reddish brown pore surface, a monomitic hyphal system with clamped generative hyphae, and broadly ellipsoid basidiospores [96]. Phylogenetically, it nests in the genus *Phlebicolorata*, in which it groups with *Phlebicolorata alboaurantia* (Figure 2).

***Phlebicolorata rosea*** (C.L. Zhao & Y.C. Dai) C.L. Zhao, comb. nov.

MycoBank: MB 843332.

Index Fungorum number: IF843332; Facesoffungi number: FoF12697.

**Basionym**—*Ceriporiopsis rosea* C.L. Zhao & Y.C. Dai, in Zhao, Wu, Liu & Dai, Nova Hedwigia 101(3–4): 409. 2015.

**Synonyms**—*Aurantiporus roseus* (C.L. Zhao & Y.C. Dai) Zmitr., Folia Cryptogamica Petropolitana (Sankt-Peterburg) 6: 100. 2018.

**Notes**—It has a poroid basidiomata with an orange-brown to reddish brown pore surface, a monomitic hyphal system having generative hyphae with clamp connections, and broadly ellipsoid basidiospores [44], which covers the characters of the genus *Phlebicolorata*. The present phylogeny supports it as a combination species inferred from the molecular evidence (Figure 2).

***Pseudophlebia*** C.L. Zhao gen. nov.

MycoBank: MB 843333.

Index Fungorum number: IF843333; Facesoffungi number: FoF12702.

Diagnosis: It is characterized by annual, resupinate to effused-reflexed basidiomata with pale ochraceous to ochraceous yellow-brown to reddish-brown hymenial surface, a monomitic hyphal system and colorless, thin-walled, smooth basidiospores. It differs from the other genus in terms of DNA sequences.

**Type species**—*Pseudophlebia setulosa* (Berk. & M.A. Curtis) C.L. Zhao.

**Etymology**—Referring to be similar to genus *Phlebia*.

Basidiomata annual, resupinate, hard, and brittle. Hymenophore is poroid or merulioid to hydnoid; the hymenial surface is pale ochraceous to ochraceous yellow-brown to reddish brown. The hyphal system is monomitic, having generative hyphae with clamp connections, IKI–, CB–; tissues unchanged in KOH. Cystidia present or not. Basidiospores are colorless, thin-walled, and smooth, IKI–, CB–.

***Pseudophlebia lindtneri*** (Pilát) C.L. Zhao, comb. nov.

MycoBank: MB 843334.

Index Fungorum number: IF843334; Facesoffungi number: FoF12699.

**Basionym**—*Peniophora lindtneri* Pilát, Bull. trimest. Soc. mycol. Fr. 53: 97. 1937.

***Pseudophlebia semisupina*** (C.L. Zhao, B.K. Cui & Y.C. Dai) C.L. Zhao, comb. nov.

MycoBank MB 843335.

Index Fungorum number: IF843335; Facesoffungi number: FoF12700.

**Basionym**—*Ceriporiopsis semisupina* C.L. Zhao, B.K. Cui & Y.C. Dai, in Zhao & Cui, Phytotaxa 164(1): 23. 2014.

***Pseudophlebia setulosa*** (Berk. & M.A. Curtis) C.L. Zhao, comb. nov.

MycoBank: MB 843336.

Index Fungorum number: IF843336; Facesoffungi number: FoF12701.

**Basionym**—*Hydnum setulosum* Berk. & M.A. Curtis, Grevillea 1 (no. 7): 100 (1873).

## 4. Discussion

In the present study, an improved classification of *Phlebia* s.l. is provided by employing seven gene phylogenetic analyses at the intergeneric level (Figure 1), which discusses the circumscription and phylogenetic relationships of genera in *Phlebia* s.l. The tree topologies are generally consistent with previous phylogenetic studies using ribosomal RNA genes (rDNA) alone or in combination with the protein-coding genes [28,31,32,33,34,38]. However, some minor differences still exist due to the different scales of sampling and lower supports for several topologies in this group.

Across all species of *Phlebia* s.l., the smooth, tuberculata, phlebioid, merulioid, odontioid–hydnoid, and poroid hymenophores are varied, such as the phlebioid species (*Phlebia radiata*), merulioid species (*Merulius tremellosus*), hydnoid species (*Mycoacia fuscoatra*), poroid species (*Ceriporiopsoides guidella*), smooth species (*Luteochaete subglobosa*), and the tuberculate species (*Phlebicolorata brevispora*). Traditionally, the hymenophore configurations of morphological study for this group play a core role, but the macromorphology of fruiting bodies and hymenophore construction did not reflect monophyletic features for this group in the present study (Figure 1), which is generally consistent with previous phylogenetic studies [32,33,34,38,43,44]. The evolution mechanism for morphology and phylogeny is still confusing mycologists. Several mycologists proposed that the transitions on the hymenophore forms have evolved multiple times in the evolution of the wood-decaying fungal groups [28,97]. 

Phylogenetically, Binder et al. [27] revealed that seven clades are found in the Polyporales viz. antrodia clade, core polyporoid clade, fragiliporia clade, gelatoporia clade, phlebioid clade, residual polyporoid clade, and the tyromyces clade. According to our results based on the combined ITS + nLSU sequence data (Figure 1), the species of *Phlebia* s.l. are nested into the phlebioid clade, which supports the previous scientific studies [31,34,38]. Three families viz. Phanerochaetaceae, Irpicaceae Spirin & Zmitr., and Meruliaceae P. Karst. were studied in the order Polyporales [97], in which the large-scale frame was put up for three families, and five genera of the family Meruliaceae were included, but the related scientific problem of *Phlebia* s.l. is still to be resolved.

Nilsson et al. revealed that in the International Nucleotide Sequence Databases, 10–21% of the 51,000 fungal ITS sequences available were annotated with incorrect taxonomic information [98]. More recently, this proportion has increased to almost 30% [99]. In the present study, we employed the type specimens and their sequences to carry out our phylogenetic tree for *Phlebia* s.l. (Figure 1 and Figure 2), which increased the reliability of sequences supporting our results of *Phlebia* s.l.

**Clade A**

Within the clade A, we recognize five subclades, with subclade I to V comprising fourteen genera as follows: Ceriporiopsoides, Climacodon, Crustodontia, Geesterania, Hydnophlebia, Hydnophanerochaete, Luteochaete, Luteoporia, Mycoacia, Mycoaciella, Odoria, Phlebiporia, Sarcodontia, and Scopuloides (Figure 1), in which all of them grouped together, as well as in the previous studies [28,34,38,97].

In subclade I, eight genera, *Crustodontia*, *Geesterania*, *Hydnophlebia*, *Luteoporia*, *Mycoaciella*, *Odoria*, *Phlebiporia*, and *Sarcodontia*, were grouped together (Figure 2), similar to a previous study’s topology [34]. The genus *Crustodontia* was proposed by Hjortstam and Ryvarden [88] to accommodate *C. chrysocreas* based on morphological data. In our analyses (Figure 2), *Crustodontia* is resolved as a monomitic hyphae system with strong support within the subclade I of Meruliaceae, in which the topology is similar to a previous study [34]. Two species, *Crustodontia nigrodontea* (C.L. Zhao & R.X. Huang) C.C. Chen & Sheng H. Wu and *C. tongxiniana* (C.L. Zhao) C.C. Chen & Sheng H. Wu, were transferred to *Crustodontia* mainly based on the phylogeny results [34]. In the present study, *Crustodontia rhododendri* is described as a new taxon of this genus based on the straw-to-ochraceous hymenial surface and the monomitic hyphal structure with clamped generative hyphae, and it groups with the type species *C. chrysocreas*. The species of *Crustodontia*, *Geesterania*, *Luteoporia*, *Mycoaciella*, and *Phlebiporia* are often classified in *Phlebia* s.l. based on their morphological similarities [14,16,31,53,58,72]. Two genera, *Geesterania* and *Phlebiporia*, nest into subclade I too, and group closely, as well as in a previous study [34]. Both genera share the similar character of turning reddish or purplish brown with KOH [72,86]. Morphologically, *Geesterania* is characterized by the dimitic hyphal system and the presence of the skeletocystidia, and additionally, it changes color when bruised or after drying [58]; phylogenetically, it groups with *Phlebiporia* based on the ITS + nLSU analysis in the previous studies [58,72], as well as the present study. *Hydnophlebia* nested within this clade as a monophyletic group with strong supports, including species from Africa, East Asia, Europe, and North America, with the typical characteristics of the membranaceous, reddish-orange basidiomata with poroid or grandinoid to hydnoid hymenophores, and, in addition, a distinctly rhizomorphic margin and a monomitic hyphal structure bearing simple-septate generative hyphae [34,100]. The monophyly of *Hydnophlebia* segregated from *Phanerochaete* s.l. was supported in the multi-gene phylogeny [28,61]. In the present study, *Hydnophlebia fissurata* nested into the genus *Hydnophlebia* and grouped with *H. canariensis* Telleria, M. Dueñas & M.P. Martín; morphologically, this species is characterized by a grandinoid hymenophore with a pale brown color, and a monomitic hyphal structure with simple-septate generative hyphae; therefore, we proposed it as a new taxon within *Hydnophlebia*. *Luteoporia* species were recorded from Asia (*Luteoporia albomarginata* F. Wu, Jia J. Chen & S.H. He, *L. citriniporia* Z.B. Liu & Yuan Yuan, and *L. lutea* (G. Cunn.) C.C. Chen & Sheng H. Wu) (China and Japan). This genus was proposed as a new genus in Polyporales based on morphological characters and molecular data [62], which is characterized by having poroid or odontioid to hydnoid hymenophores with golden yellow basidiomata turning purple with KOH [34,62]. In the present study, a new species *Luteoporia straminea* was found in Yunnan Province and is characterized by having odontioid, straw to pale orange hymenophores, and tissue that becomes reddish in KOH, and phylogenetically, it is sister to *L. lutea*, with high statistical supports (Figure 2). Four species inside *Luteoporia* having a different macroscopical hymenophore with poroid or odontioid to hydnoid eye-attracting characters are phylogenetically clustered together closely within the genus *Luteoporia*, which suggests that the easily observing macroscopical hymenophore characters and the phylogenetical topology results are not similar; therefore, it needs deeper studies in the future. The genus *Mycoaciella* formed a monophyletic lineage (Figure 2), which is similar to the related research [34]. It was considered a synonym of *Phlebia* by several studies [14,76], but it was recently accepted as a separate genus [34,101]. In this study, *Mycoaciella brunneospina* grouped with *M. bispora*, but the morphological characters show that it has a monomitic hyphal system, which expands the generic concept extension. *Odoria* (Meruliaceae, Basidiomycota) was described as a new genus established for the threatened old-growth forest polypore *Phaeolus alborubescens*; morphologically, it has the special character of the pileate, with sappy basidiomata and thick-walled basidiospores [66], in which the morphological characteristics imply that it is incompatible with similar topology members from the molecular analysis. *Sarcodontia* is an old genus that was established in 1866 [102] with the type species of *S. crocea* (Schwein.) Kotl., which is unstable in phylogenetic analysis, perhaps for the lower supports. Recently, the separation of these genera from *Phlebia* s.l. has been supported by several mycologists mainly based on the phylogenetic characteristics [34,58], and our multi-gene phylogenetic analyses also supported this (Figure 2). Due to possessing variable hymenophore configurations, *Phlebia* s.l. is still highly polyphyletic with members distributed in three families viz. Phanerochaetaceae, Irpicaceae, and Meruliaceae [38,44], which reveal that the transitions between hymenophore or basidiocarp forms have evolved multiple times in the evolution of the phlebioid clade [28,97]; therefore, the molecular methods perhaps divide traditional taxonomic genera into several smaller genera.

In subclade II, three genera viz. *Climacodon*, *Luteochaete*, and *Scopuloides* formed a monophyletic lineage, in which the topology is similar to the previous studies [34,38,58]. The genus *Climacodon* originally was located in the family Climacodontaceae, but this family was a synonym of Meruliaceae; therefore, this genus was treated as a member of Meruliaceae [38], in which it grouped with *Ceriporiopsis guidella* and *C. lagerheimii* with low supports. The genus *Luteochaete* was established to accommodate *Phanerochaete subglobosa* Sheng H. Wu, which has subceraceous to coriaceous basidiocarps with a smooth hymenophore turning greenish yellow in KOH, simple-septate hyphae, and broadly ellipsoid or subglobose basidiospores [34], and it is resolved as a monophyletic group with strong supports (Figure 2), as in a previous study [34]. *Phlebia wuliangshanensis* from Yunnan Province was conspecific with *L. subglobosa* [34], even though the specimens from both taxa are distant, perhaps all of them have the similar latitude and ecological environment. *Scopuloides* formed a well-supported group within *Phlebia* s.l. in the studies [34,38,58,66], and our molecular study also showed that it was a sister to *Climacodon* with lower supports (Figure 2).

In subclade III, the genus *Ceriporiopsoides* formed a single lineage with lower supports (Figure 2) with a similar topology to a previous study [34]. *Ceriporiopsis guidella* Bernicchia & Ryvarden and *C. lagerheimii* Læssøe & Ryvarden grouped together and formed a monophyletic lineage, in which both taxa nested among species of *Ceriporiopsis* and *Phlebia* solely [34,43,53,64]; morphologically both species have the unique characters of hard, brittle, cartilaginous basidiomata; therefore, in the present study, we propose a new genus *Ceriporiopsoides* to accommodate both species based on the morphological characters and molecular phylogeny (Figure 2). 

In subclade IV, *Hydnophanerochaete* was recently built to comprise the type species *H. odontoidea* (Sheng H. Wu) Sheng H. Wu & C.C. Chen [60], and this species was originally described under the genus *Phanerochaete* [103]. According to the present molecular data, it nests into *Phlebia* s.l. (Figure 2) as in a similar phylogenetic result [34]. *Phlebia ailaoshanensis* was described from Yunnan, China, which was synonymized under *H. odontoidea*, even though the presence of little morphological differences; in addition, both species have overlapping geographic distributions in eastern Asia.

In subclade V, *Mycoacia* forms a single lineage (Figure 2) in the current study, and it was used to accommodate *Phlebia*-like species covering the characters of the odontioid to hydnoid hymenophore and a monomitic hyphal system [104], but later it was treated as a synonymy with *Phlebia* [13]. Our phylogenetical analysis (Figure 2) suggests that it is a monophyletic genus, including the generic type species *M. fuscoatra*, as well as the previous study [34]. *Ceriporiopsis gilvescens* (Bres.) Domański is the type species of *Ceriporiopsis* Domański, and it was transferred to the genus *Mycoacia* as *M. gilvescens* (Bres.) Zmitr. [105] based on the phylogenetic analysis, but the lack of the type species of *Ceriporiopsis* makes it difficult to resolve the new location, so the taxonomy research about the species of *Ceriporiopsis* needs to arrange a schedule for mycologists.

**Clade B**

In subclade VI, *Phlebia* s.s. taxa grouped closely with *Pseudophlebia* [31,34,38], which is similar to our present analysis result (Figure 1 and Figure 2). Due to the whole of *Phlebia* s.l. being highly polyphyletic, many species of *Phlebia* s.l. have been presently placed in other genera based on morphological and molecular characteristics. Therefore, the core taxa of *Phlebia* s.s. comprise *P. acerina* Peck, *P. floridensis* Nakasone & Burds, *P. radiata*, and *P. rufa* (Pers.) M.P. Christ. based on the molecular evidence. In the present study, *Phlebia niveomarginata* and *P. poroides* cluster into *Phlebia* s.s. (Figure 2), so we propose both of them to be new species. Four species *Aurantiporus mayaensis* (Ginns, D.L. Lindner & T.J. Baroni) Zmitr., *Ceriporiopsis semisupina* C.L. Zhao, B.K. Cui & Y.C. Dai, *Phlebia lindtneri* (Pilát) Parmasto, and *P. setulosa* (Berk. & M.A. Curtis) Nakasone grouped closely and formed a lone lineage [34] which is similar to this study (Figure 2); additionally, all of them have the morphological characters of hard and brittle basidiomata with the pale ochraceous to ochraceous yellow-brown to reddish brown hymenial surface and mostly present cystidia; therefore, we describe a new genus *Pseudophlebia* to comprise them based on the morphological and molecular evidence.

In subclade VII, two genera *Pappia* and *Phlebicolorata* grouped together within this clade (Figure 2), but *Pappia* formed a single lineage, and morphologically, *Pappia* is characterized by the soft, pileate basidiomata with poroid hymenopores and the presence of chalmydospores, which is inconsistent with the species of *Phlebicolorata* [105]. *Ceriporiopsis alboaurantia* C.L. Zhao, B.K. Cui & Y.C. Dai was originally reported on the genus *Ceriporiopsis* based on the poroid basidiomata with a monomitic hyphal system with clamped generative hyphae and thin-walled basidiospores [43], in which it was sister to *C. pseudoplacenta* Vlasák & Ryvarden closely as well as in the study [64]. The species *Ceriporiopsis rosea* C.L. Zhao & Y.C. Dai was described within *Ceriporiopsis*, and it grouped with a clade comprising *C. alboaurantia* and *C. pseudoplacenta* [70]. *Aurantiporus croceus* (Pers.) Murrill was sister to *C. pseudoplacenta* [38]; four species *Aurantiporus croceus*, *Ceriporiopsis alboaurantia*, *C. pseudoplacenta,* and *C. rosea* grouped together closely and isolated from *Aurantiporus* or *Ceriporiopsis* [34] as well as the present study (Figure 2); therefore, we propose a new genus of *Phlebicolorata* to accommodate them based on the morphological and molecular examination.

**Clade C**

In subclade VIII, *Merulius* formed a single lineage with a lower support (Figure 2), which is a different topology from a previous study [34]. The genus *Merulius*, typified by *M. tremellosus* Schrad., is characterized by the typical merulioid hymenophore, and it was considered a synonym of *Phlebia* based on morphological evidence [10], and due to the lack of enough morphological and molecular data, the previous study [34] put this genus inside *Phlebia* s.l. In the present study, we suggest this genus as a monophyletic genus based on the evidence of the morphological and molecular data (Figure 2); in addition, the Index Fungorum (accessed on March 6, 2022) registers 525 records; therefore, it refrains from making more name changes. The species *Merulius sinensis* nests into the genus of *Merulius* and is a sister to *M. nantahaliensis* (Nakasone & Burds.) C.L. Zhao, so we propose it as a new species.

**Clade D**

In subclade IX, *Phlebia centrifuga* P. Karst. was found on a fallen trunk of *Abies excelsa* in Finland (1881), which is easily recognized by the characteristics of densely and irregularly papillose and partly radially or unevenly wrinkled hymenophore with whitish, fibrillose–strigose margin, and it was marginalized in the phylogenetical tree [34,38] as well as the present study (Figure 2), so a new genus of *Hermanssonia* Zmitr. was established [105].

*Phlebia* s.l. species are an extensively studied group, distributed worldwide [7,8,13,14,15,16,17,27,28,29,30,32,33,34,106,107] and mainly found on hardwood, although a few species grow on coniferous wood [7,106]. Many species of *Phlebia* s.l. were found in America, Asia, and Europe, but most of them did not record in Africa and Oceania, in which we presumed that the specimens of *Phlebia* s.l. were undersampled by the mycologists. Research on the new taxa related to wood-decaying fungi of *Phlebia* s.l. from China have been reported [31,32,33,43,64,70,106], in which twelve *Phlebia* s.l. species were reported as new taxa recently. The macromorphology of fruiting bodies and hymenophore construction did not reflect the monophyletic result; therefore, focusing on the relationships between the host and *Phlebia* s.l. species may be very interesting in further deep studies. The studies on the molecular systematics of *Phlebia* s.l. also push the development of the Tree of Life, which will be useful to push further research on fundamental research and applied research of fungi. More and more species of *Phlebia* s.l. are found in subtropical and tropical Asia, especially they are reported in tropical China recently [3,4,106], and it is proved that this area has a unique ecological environment and many mycologists carry out studies in tropical Asia [4,30,31,32,33,44,64,106].

In conclusion, our study conducted a basic survey on species diversity, generic recognition, and phylogeny of *Phlebia* s.l. (Meruliaceae) of Polyporales, especially in China, with many taxa sampling and much sequence data from ITS, nLSU, TEF1, mt-SSU, GAPDH, RPB1, and RPB2. Within *Phlebia* s.l., we overall recognize 20 genera, including three new ones, describing seven new species, and propose 17 new combinations. The status of some recognized genera and species can be further evaluated by phylogenetic or phylogenomic analyses based on more taxa and sequences. Based on the historical reason and the normal morphological characters of *Phlebia* s.l., so many similar taxa got together in this genus, and our present study is another brick in the wall of a house, which needs more and more mycologists to reel silk from cocoons and lift the veil for this genus in the future. Most of our new species in *Phlebia* s.l. were found in Yunnan Province, southwest China, a similar phenomenon was found in other groups of wood-rotting fungi [108,109,110,111,112]. The area is a hotspot for biodiversity, and more new taxa will be discovered after further investigations.

## Figures and Tables

**Table 1:** Primers used in this study, with sequences and references.

Locus	Primer	Primer Sequences 5′ to 3′	Annealing Temperature	Orientation	References
internal transcribed spacer (ITS)	ITS5	GGA AGTAAA AGT CGTAACAAGG	58	Forward	[45]
	ITS4	TCCTCCGCTTATTGATATGC	58	Reverse	[45]
large subunit ribosomal DNA (LSU)	LR0R	ACCCGCTGA ACTTAAGC	48	Forward	[45]
	LR7	TACTACCACCAAGATCT	48	Reverse	[45]
translation elongation factor 1-alpha (*tef 1-α*)	EF1-983F	GCYCCYGGHCAYCGTGAYTTYAT	60	Forward	[47]
	EF1-2218R	ATGACACCRACRGCRACRGTYTG	60	Reverse	[47]
small subunit of mitochondrial rRNA gene (mtSSU)	MS1	CAGCAGTCAAGAATATTAGTCAATG	52	Forward	[45]
	MS2	GCGGATTATCGAATTAAATAAC	52	Reverse	[45]
Glyceraldehyde 3-phosphate dehydrogenase (GAPDH)	GAPDH-F	ATGGTCTACATGTTCAAGTACGAC	50	Forward	[48]
	GAPDH-R	TCGACGAGGGGATGATGT T	50	Reverse	[48]
RNA polymerase II largest subunit (rpb1)	RPB1-Af	GARTGYCCDGGDCAYTTYGG	60	Forward	[49]
	RPB1-Cr	CCNGCDATNTCRTTRTCCATRTA	60	Reverse	[49]
RNA polymerase II second largest subunit (*rpb2*)	bRPB2-6F	TGGGGYATGGTNTGYCCYGC	52	Forward	[50]
	bRPB2-7.1R	CCCATRGCYTGYTTMCCCATDGC	52	Reverse	[50]

**Table 2:** Names, voucher codes, and corresponding GenBank accession numbers of sequences used in this study.

Species Name	Sample No.	GenBank Accession No.							References
		ITS	nLSU	RPB1	RPB2	TEF1	GAPDH	mt-SSU	
*Antrodiella stipitate*	FD-136	KP135314	KP135197	KP134886	—	—	—	—	[28]
*Bondarzewia montana*	AFTOL-ID 452	DQ200923	DQ234539	DQ256049	AY218474	DQ059044	—	—	[51]
*Ceriporia viridans*	GC 1708-211	LC427027	LC427049	LC427062	—	—	—	—	[52]
*Ceriporiopsis aneirina*	Dai 12657	KF845952	KF845945	—	—	—	—	—	[43]
*C. pseudogilvescens*	Cui 6824	KU509523	—	—	—	—	—	—	[53]
*C. resinascens*	BRNM 686416	FJ496679	FJ496703	—	—	—	—	FJ496737	[41]
*Ceriporiopsoides guidella*	HUBO 7659	FJ496687	FJ496722	—	—	—	—	FJ496740	[41]
*C. lagerheimii*	Dai 12304	KX161647	KX161651	—	—	—	—	—	Unpublished
*Cerrena unicolor*	FD-299	KP135304	KP135209	KP134874	KP134968	—	—	—	[28]
*Climacocystis borealis*	FD-31	KP135308	KP135210	KP134882	KP134895	—	—	—	[28]
*Climacodon septentrionalis*	AFTOL-767	AY854082	AY684165	AY864873	AY780941	AY885151	—	—	Unpublished
*C. septentrionalis*	CBS 131.40	MH856064	MH867555	—	—	—	—	—	[54]
*C. septentrionalis*	FP-72067	KP135345	—	—	—	—	—	—	[28]
*C. septentrionalis*	RLG-6890-Sp	KP135344	—	—	—	—	—	—	[28]
*Coriolopsis caperata*	CR 22	JN164999	JN164789	—	—	—	—	—	[55]
*Crustodontia chrysocreas*	HHB-6333-Sp	KP135358	KP135263	KP134861	KP134908	—	—	—	[28]
*C. chrysocreas*	FCUG2827	HQ153411	—	—	—	—	—	—	[56]
*C. nigrodontea*	CLZhao 2729	MT896823	MT896819	ON960280 *	—	ON892520 *	—	—	[33]; Present study
*C. nigrodontea*	**CLZhao 2758**	MT896824	—	—	—	—	—	—	[33]
*C. rhododendri*	CLZhao 851	MW732399	MW724791	ON942236 *	ON918559 *	—	—	MW732759	Present study
*C. rhododendri*	CLZhao 6168	MW732400	MW724792	ON950240 *	—	ON892523 *	ON892530 *	MW732760	Present study
*C. rhododendri*	CLZhao 16995	MW732396	MW724788	—	—	—	—	MW732768	Present study
*C. taiwanensis*	GC 1703-88	MZ636944	MZ637106	MZ748466	OK136049	—	—	—	[28]
*C. taiwanensis*	Wu 9310-21	MZ636945	MZ637107	—	—	—	—	—	[34]
*C. tongxiniana*	CLZhao 2255	MT020773	MT020751	—	—	—	—	—	[31]
*C. tongxiniana*	CLZhao 5217	MT020778	MT020756	ON892526 *	ON918558 *	ON892521 *	—	MW732754	[31]; Present study
*C. uda*	FP-101544-Sp	KP135361	KP135232	KP134859	KP134909	MZ913649	—	—	[57]
*Daedalea quercina*	FP56429	KY948809	KY948883	KY948989	—	—	—	—	[38]
*Earliella scabrosa*	PR1209	JN165009	JN164793	JN164819	JN164866	JN164894	—	—	[55]
*Efibula americana*	FP-102165	KP135016	KP135256	KP134808	KP134916	—	—	—	[28]
*E. tuberculate*	OM-6707	KP135017	—	KP134807	—	—	—	—	[28]
*Fomitopsis pinicola*	AFTOL-770	AY854083	AY684164	AY864875	AY786056	AY885152	—	FJ436112	Unpublished
*Fragiliporia fragilis*	Dai 13080	KJ734260	KJ734264	—	KJ790248	KJ790245	—	KJ734268	[44]
*F. fragilis*	Dai 13559	KJ734261	KJ734265	—	KJ790249	KJ790246	—	KJ734269	[44]
*F. fragilis*	Dai 13561	KJ734262	KJ734266	—	KJ790250	KJ790247	—	KJ734270	[44]
*Ganoderma lingzhi*	Cui-9166	MG732955	—	—	—	MH127978	—	—	Unpublished
*Geesterania carneola*	MCW 388/12	KY174999	KY174999	—	KY175011	KY175013	—	—	[58]
*G. carneola*	SP 446193	NR_158508	—	—	—	—	—	—	[58]
*G. davidii*	MCW 396/12	KY174998	KY174998	—	KY175012	KY175016	—	—	[58]
*Gelatopori subvermispora*	FD-354	KP135312	KP135212	KP134879	KP134961	—	—	—	[28]
*Grammothelopsis subtropica*	Cui 9041	JQ845096	JQ845099	—	—	—	—	—	Unpublished
*Hermanssonia centrifuga*	CBS 125890	MH864088	MH875547	—	—	—	—	—	[54]
*H. centrifuga*	HHB-9239-Sp	KP135380	KP135262	KP134844	KP134974	MZ913721	—	—	[28]
*Heterobasidion annosum*	VL-296	JF440572	—	—	—	—	—	—	[59]
*Hydnophanerochaete odontoidea*	CLZhao 3996	MH784926	MH784936	—	—	—	—	—	[30]
*H. odontoidea*	CLZhao 4036	MH784927	MH784937	—	—	—	—	—	[30]
*H. odontoidea*	Wu 9310-29	LC379002	—	—	—	—	—	—	[60]
*H. odontoidea*	TNM: GC 1308-45	LC363486	LC363492	LC363497	—	—	—	—	[60]
*H. odontoidea*	TNM: Chen 1376	LC363485	LC363491	LC363496	—	—	—	—	[60]
*Hydnophlebia acanthocystis*	FP 150571	KY948767	KY948844	KY948914	—	—	—	—	[38]
*H. canariensis*	MA-Fungi 86619	KF483009	KF528100	—	—	—	—	—	[61]
*H. caspica*	FCUG3159	HQ153410	—	—	—	—	—	—	[56]
*H. chrysorhiza*	FD-282	KP135338	KP135217	KP134848	KP134897	—	—	—	[28]
*H. fimbriata*	Dai 11672	KJ698633	KJ698637	—	—	—	—	—	[44]
*H. fissurata*	CLZhao 2900	MW732402	MW724794	ON892527 *	ON892536 *	ON968926 *	—	MW732762	Present study
*H. gorgonea*	MA-Fungi 86642	KF483031	KF528122	—	—	—	—	—	[61]
*H. omnivora*	KKN-112-Sp	KP135334	KP135216	KP134846	—	—	—	—	[28]
*Hyphoderma setigerum*	FD-312	KP135297	KP135222	KP134871	—	—	—	—	[28]
*Hypochnicium bombycinum*	HHB-12631-sp	KY948801	—	KY948930	—	—	—	—	[38]
*Junghuhnia nitida*	CBS 459.50	MH856708	MH868226	—	—	—	—	—	[54]
*Lopharia cinerascens*	FP-105043-sp	JN165019	JN164813	—	—	—	—	—	[55]
*Luteochaete subglobosa*	CLZhao 3475	MK881897	MK881787	—	—	—	—	—	Unpublished
*L. subglobosa*	CLZhao 3645	MK881899	MK881789	—	—	ON892522 *	—	MW732758	Present study
*L. subglobosa*	GC 1605-4	MZ636995	MZ637156	MZ748455	OK136053	MZ913645	—	—	[34]
*L. subglobosa*	Wu 870918	MZ636996	GQ470662	MZ748456	OK136054	MZ913646	—	—	[34]
*Luteoporia albomarginata*	Dai 15229	KU598873	KU598878	—	—	—	—	—	[62]
*L. albomarginata*	GC 1702-1	LC379003	LC379155	LC379160	LC387358	LC387377	—	—	[60]
*L. citriniporia*	Dai 19507	MT872218	MT872216	—	—	—	—	—	[63]
*L. lutea*	GC 1409-1	MZ636998	MZ637158	MZ748467	OK136050	MZ913656	—	—	[34]
*L. straminea*	CLZhao 5794	OM897115 *	OM897114 *	—	—	—	—	—	Present study
*L. straminea*	CLZhao 18947	MW732407	MW724799	—	—	—	—	MW732765	Unpublished
*Merulius fuscotuberculata*	CLZhao 10227	MT020759	MT020737	ON892524 *	ON892537 *	ON855009 *	ON634683 *	—	Present study
*M. fuscotuberculata*	CLZhao 10239	MT020760	MT020738	ON892525 *	ON892538 *	ON936910 *	ON980563 *	—	Present study
*M. giganteus*	FP-135344-Sp	KP135307	KP135228	—	—	—	—	—	[28]
*M. hydnoidea*	HHB 1993sp	KY948778	KY948853	KY948921	—	—	—	—	[38]
*M. nantahaliensis*	HHB 2816sp	KY948777	KY948852	KY948920	—	—	—	—	[38]
*M. sinensis*	CLZhao 2562	MW732401	MW724793	—	—	—	ON892532 *	MW732761	Present study
*M. tomentopileata*	CLZhao 5833	MT020761	MT020739	—	—	—	—	—	[31]
*M. tomentopileata*	CLZhao 10274	MT020771	MW732469	—	—	—	ON892531 *	MW732752	[31]; Present study
*M. tremellosus*	CBS 217.56	MH857589	MH869138	—	—	—	—	—	[54]
*M. tremellosus*	FBCC278	LN611126	LN611126	—	LN611035	—	LN611072	—	[48]
*Mycoacia aurea*	FCUG 2767	HQ153409	—	—	—	—	—	—	[56]
*M. aurea*	RLG 5075sp	KY948759	—	KY948918	—	—	—	—	[38]
*M. fuscoatra*	HHB-10782-Sp	KP135365	KP135365	KP134857	KP134910	—	—	—	[28]
*M. fuscoatra*	OMC 1380	KY948754	—	—	—	—	—	—	[38]
*M. gilvescens*	BRNM 710166	FJ496684	FJ496720	—	—	—	—	—	[41]
*M. gilvescens*	Chen 156	MZ636935	MZ637098	—	—	—	—	—	[34]
*M. gilvescens*	Chen 3340	MZ636936	MZ637099	MZ748446	OK136039	MZ913651	—	—	[34]
*M. gilvescens*	Yuan 2752	KF845953	KF845946	—	—	—	—	—	[43]
*M. kunmingensis*	CLZhao 152	KX081072	KX081074	—	—	—	—	—	[64]
*M. kunmingensis*	CLZhao 153	KX081073	KX081075	—	—	—	—	—	[64]
*M. livida*	FP 135046 sp	KY948758	KY948850	KY948917	—	—	—	—	[38]
*M. livida*	FBCC 1283	LN611123	LN611123	—	LN611033	—	—	—	[48]
*M. nothofagi*	HHB-4273-Sp	KP135369	KP135266	KP134858	KP134911	—	—	—	[28]
*M. nothofagi*	HHB-6906-Sp	KP135368	—	—	—	—	—	—	[28]
*M. subfascicularis*	Chen 3873	MZ637007	MZ637168	—	—	—	—	—	[34]
*M. subfascicularis*	Wu 1004-11	MZ637008	—	MZ748448	OK136044	MZ913653	—	—	[34]
*M. tuberculata*	MG 128	HQ153425	—	—	—	—	—	—	[56]
*M. tuberculata*	FCUG 3186	HQ153418	—	—	—	—	—	—	[56]
*Mycoaciella bispora*	EL 13_99	AY463446	AY586692	—	—	—	—	—	[40]
*M. brunneospina*	CLZhao 15876	MW732404	MW724796	ON892515 *	—	—	—	MW732764	Present study
*Obba rivulosa*	FP-135416-Sp	KP135309	KP135208	KP134878	KP134962	—	—	—	[28]
*O. valdiviana*	FF484	HQ659236	—	—	—	—	—	—	[65]
*Odoria alborubescens*	BP 106943	MG097864	MG097867	MG213724	MG213723	—	—	—	[66]
*O. alborubescens*	BRNU 627479	JQ821319	JQ821318	—	—	—	—	—	[67]
*Panus fragilis*	HHB-11042-Sp	KP135328	KP135233	KP134877	KP134970	—	—	—	[28]
*Pappia fssilis*	814	HQ728291	HQ729001	—	—	—	—	—	[68]
*P. fssilis*	BRNM 699803	HQ728292	HQ729002	—	—	—	—	—	[68]
*Perenniporia medulla-panis*	Cui 14515	MG847214	MG847223	—	—	—	—	—	Present study
*Perenniporiella neofulva*	MUCL 45091	FJ411080	FJ393852	—	—	—	—	—	[69]
*Phanerochaete laevis*	HHB 15519	KP135149	KP135249	KP134836	KP134952	—	—	—	[28]
*P. rhodella*	FD-18	KP135187	KP135258	KP134832	KP134948	—	—	—	[28]
*P. sanguinea*	HHB-7524	KP135101	KP135244	KP134825	KP134943	—	—	—	[28]
*P. velutina*	CBS 412.50	MH856692	MH868209	—	—	—	—	—	[54]
*Phlebia acerina*	FD-301	KP135378	KP135260	KP134862	—	—	—	—	[28]
*P. acerina*	DR 60sp	KY948773	—	KY948924	—	—	—	—	[38]
*P. albida*	GB 1833	KY948748	KY948889	KY948960	—	—	—	—	[38]
*P. griseoflavescens*	MR-4310	KY948797	KY948888	KY948963	—	—	—	—	[38]
*P. floridensis*	HHB-9905	KP135383	KP135264	KP134863	KP134899	—	—	—	[28]
*P. floridensis*	FP 102562T	KP135386	—	—	—	—	—	—	[28]
*P. leptospermi*	CBS 126031	MH863894	MH875355	—	—	—	—	—	[54]
*P. niveomarginata*	CLZhao 18972	MW732409	MW724801	ON892518 *	ON925000 *	ON892529 *	ON892519 *	—	Present study
*P. niveomarginata*	CLZhao 19089	MW732410	MW724802	—	—	—	ON892535 *	—	Present study
*P. ochraceofulva*	FBCC 360	LN611117	LN611117	—	LN611028	—	LN651203	—	[48]
*P. poroides*	CLZhao 16121	MW732405	MW724797	ON892516 *	ON918560 *	—	ON892533 *	—	Present study
*P. poroides*	CLZhao 18421	MW732406	MW724798	ON892517 *	ON924999 *	ON892528 *	ON892534 *	—	Present study
*P. queletii*	CBS 234.56	MH857600	MH869148	—	—	—	—	—	[54]
*P. radiata*	CBS 285.56	MH857642	MH869187	—	—	—	—	—	[54]
*P. radiata*	FBCC 1376	LN611102	LN611102	—	LN611014	—	LN611061	—	[48]
*P. rufa*	CBS 213.47	MH856224	MH867751	—	—	—	—	—	[54]
*P. rufa*	HHB-14924	KP135374	—	—	—	—	—	—	[48]
*Phlebicolorata alboaurantia*	**Cui 4136**	KF845955	KF845948	—	—	—	—	—	[43]
*P. brevispora*	FBCC 1463	LN611135	LN611135	—	LN611041	—	LN611081	—	[48]
*P. crocea*	Miettinen-16483	KY948745	KY948901	KY948927	—	—	—	—	[38]
*P. pseudoplacenta*	Miettinen 18997	KY948744	KY948902	KY948926	—	—	—	—	[38]
*P. rosea*	Dai 13584	KJ698636	KJ698640	—	—	—	—	—	[70]
*P. rosea*	Dai 13573	KJ698635	KJ698639	—	—	—	—	—	[70]
*Phlebiopsis castanea*	He 3249	MT386375	—	—	—	—	—	—	Unpublished
*P. gigantea*	FP-70857	KP135390	KP135272	KP134821	KP134930	—	—	—	[28]
*Phlebiporia bubalina*	Dai 9798	KY131842	KY131901	—	—	—	—	—	[71]
*P. bubalina*	Dai 13168	KC782526	KC782528	—	—	—	—	—	[72]
*P. bubalina*	Dai 15179	KY131843	KY131902	—	—	—	—	—	[71]
*Piptoporus betulinus*	L-15603-Sp	KC585373	KC585202	KY949005	—	—	—	—	[73]
*Podoscypha parvula*	CBS 331.66	JN649361	JN649361	—	—	—	—	—	[74]
*Polyporus squamosus*	AFTOL-704	DQ267123	AY629320	DQ831023	DQ408120	DQ028601	—	JN710743	Unpublished
*Pseudophlebia lindtneri*	GB 501	KY948772	KY948847	KY948923	—	—	—	—	[38]
*P. mayaensis*	JV 1504/128	KT156706	—	—	—	—	—	—	Unpublished
*P. mayaensis*	TJB 10228	HM772140	HM772139	—	—	—	—	—	[75]
*P. semisupina*	Cui 10222	KF845956	KF845949	—	—	—	—	—	[43]
*P. setulosa*	HHB-6891-Sp	KP135382	KP135267	KP134864	KP134901	MZ913650	—	—	[28]
*P. setulosa*	PH 11749	GU461312	GU461312	—	—	—	—	—	[76]
*Rhizochaete americanus*	FP-102188	KP135409	KP135277	KP134815	KP134934	—	—	—	[28]
*R. radicata*	FD-123	KP135407	KP135279	KP134816	KP134937	—	—	—	[28]
*R. rubescens*	Wu 0910-45	LC387335	MF110294	LC387348	LC387370	LC270925	—	—	[60]
*Sarcodontia crocea*	BRNM 721609	KX831470	KX831472	—	—	—	—	—	[77]
*S. crocea*	OMC 1488	KY948798	KY948903	KY948928	—	—	—	—	[38]
*Scopuloides allantoidea*	GC 1602-11	MZ637080	MZ637278	—	—	—	—	—	[34]
*S. dimorpha*	FP-102935-Sp	KP135353	KP135285	KP134855	KP134905	—	—	—	[28]
*S. hydnoides*	FP-150473	KP135355	KP135284	KP134854	—	—	—	—	[28]
*S. rimosa*	HHB-7042-Sp	KP135350	KP135282	KP134853	KP134903	—	—	—	[28]
*S. rimosa*	HHB-15484-Sp	KP135352	KP135281	KP134851	KP134902	MZ913665	—	—	[28]
*S. rimosa*	RLG-5104-Sp	KP135351	KP135283	KP134852	KP134904	—	—	—	[28]
*Sebipora aquosa*	Dai 13592	KU376422	KX161660	—	—	—	—	—	Unpublished
*Skeletocutis chrysella*	FD-305	KP135310	KP135286	KP134890	KP134976	—	—	—	[28]
*S. nivea*	Miettinen-9950	KY953045	KY953045	KY948969	—	—	—	—	[78]
*S. odora*	L-13763-sp	KY948830	KY948893	KY949046	—	—	—	—	[38]
*Steccherinum ochraceum*	KHL11902	JQ031130	JQ031130	—	—	—	—	—	[27]
*Stereum hirsutum*	FPL-8805	AY854063	AF393078	AY864886	—	AY885159	—	—	Unpublished
*Trametes suaveolens*	Cui 11568	KR605823	KR605766	—	—	—	—	—	[79]
*Tyromyces chioneus*	FD-4	KP135311	KP135291	KP134804	KP134977	—	—	—	[28]
*T. galactinus*	L-15951-sp	KY948829	KY948892	KY948966	—	—	—	—	[38]
*Xanthoporus syringae*	Gothenburg 1488	JN710607	—	—	—	—	—	—	[65]

Type specimens are indicated in bold. The new strains are indicated by an asterisk (*).

## Data Availability

All sequence data generated for this study can be accessed via GenBank: https://www.ncbi.nlm.nih.gov/genbank/ (accessed on 17 August 2022). The sequence alignments were deposited in TreeBase (ID 28428).
